# Bottleneck size drives the evolution of cooperative traits in an aggregative multicellular myxobacterium

**DOI:** 10.1371/journal.pbio.3003499

**Published:** 2026-01-06

**Authors:** Jyotsna Kalathera, Prakhar Jaiswal, Neha Mandal, Vishwa Patel, Vishwesha Guttal, Sandeep Krishna, Samay Pande

**Affiliations:** 1 Bacterial Ecology and Evolution group, Department of Microbiology and Cell Biology, Indian Institute of Science, Bengaluru, India; 2 Theoretical Ecology and Evolution laboratory, Centre for Ecological Sciences, Indian Institute of Science, Bengaluru, India; 3 Simons Centre for the Study of Living Machines, National Centre for Biological Sciences-TIFR, Bengaluru, India; Wageningen University, NETHERLANDS, KINGDOM OF THE

## Abstract

Repeated population bottlenecks influence the evolution and maintenance of cooperation. However, it remains unclear whether bottlenecks select all cooperative traits expressed by an organism or only a subset of them. *Myxococcus xanthus*, a social bacterium, displays multiple cooperative traits, including growth, predation, sporulation in multicellular fruiting bodies, and germination. Using laboratory evolution experiments, we investigated the effect of repeated stringent versus relaxed population bottlenecks on the evolution of these four cooperative traits when they were all under selection. We found that only fruiting body formation and growth were positively selected under the stringent regimen, while the other two traits were selected against. The pattern was reversed in the relaxed regimen. Populations propagated under the relaxed regimen also exhibited greater fitness across the entire life cycle and maintained higher trait variations, including coexistence of cooperative and exploitative strategies. Genomic analyses identified mutations in σ^54^ interacting protein and DNA binding response regulator protein associated with adaptations in stringent and relaxed regimens, respectively. Furthermore, similar trade-offs, for example, between sporulation and germination, are also seen among natural populations of *M. xanthus*. Overall, we demonstrate that different bottleneck sizes drive the evolution of cooperative life history traits in distinct ways, often via trade-offs that constrain their joint optimization.

## Author summary

Population bottlenecks have long been known to stabilize cooperation. However, we show that repeated bottlenecks affect different cooperative traits in distinct ways. Using an experimental evolution approach to study the effect of population bottleneck sizes on four social traits in *Myxococcus xanthus*—sporulation, germination, predation, and growth—we show that stringent bottlenecks favor sporulation and growth, while relaxed bottlenecks instead promote predation and germination. Relaxed bottlenecks maintained heterogeneity with coexisting cooperators and exploiters, while stringent bottlenecks produced homogeneous cooperator populations. We also identify mutations in key regulatory proteins driving these adaptations. Furthermore, we show that such trade-offs between social traits are common in natural populations of *M. xanthus*. Taken together, we demonstrate how population bottlenecks shape the evolution of life history strategies in *M. xanthus*.

## Abstract 3

Population bottlenecks shape the evolution of cooperative traits in Myxococcus xanthus through life cycle trade-offs. This study shows that stringent bottlenecks favor growth and sporulation, while relaxed bottlenecks favor predation and germination, revealing how distinct evolutionary pressures influence cooperation across different environmental contexts.

## Introduction

Examples of cooperative microbial interactions in which cells help their clonemates are abundant [1–3]. However, explaining the evolution and stability of such interactions is challenging [4,5]. A significant fraction of cooperative interactions is driven by diffusible “public good” molecules that are susceptible to exploitation by non-producers [6–9], as they benefit from the availability of freely available public goods produced by cooperators in the environment without contributing resources towards their production [5,10–13].

Population bottlenecks can stabilize cooperation. In lab evolution experiments, repeated stringent bottlenecks have been shown to select biofilm formation [14] and multicellular development in both prokaryotic and eukaryotic microbes [15,16]. This phenomenon is attributed to the purging of non-cooperating variants [15,17–22]. We observed that for most studies that demonstrate the effects of population bottlenecks on the evolution and maintenance of cooperation, the model organism could potentially express multiple social traits [6,14–16,23]. However, the focus of investigation has generally been limited to only one or a minority of the subset of these social traits. Since the expression of multiple social traits is common [24–26], it is crucial to investigate whether stringent bottlenecks positively influence the stability of all or only a subset of cooperative traits that a microbe can express.

Stringent bottlenecks reduce diversity, purge non-cooperators, and limit conflict [16,27]. If a variant that efficiently expresses a cooperative trait survives such bottlenecks [27,28], it is likely to become fixed in the population and promote higher relatedness in the successive expansion of the population [1]. In contrast, relaxed bottleneck events can maintain extant diversity and hence result in clonal interference that favors strategies where variants can reap maximum benefits without investing resources [29,30]. This might create an opportunity for the evolution of cheaters that exploit cooperators expressing costly social traits, as reduced relatedness allows for the persistence of such exploitative behavior [22].

**Fig 1:**
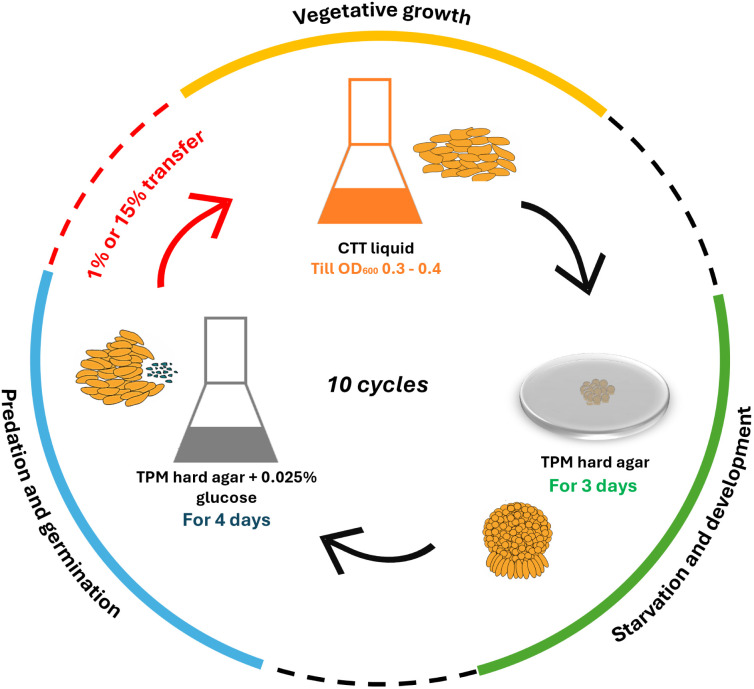
Outline of the life cycle experimental evolution of *M. xanthus* with either 1% (stringent) or 15% (relaxed) population bottlenecks. Four different colonies of *M. xanthus* (GV1) were used to establish four parallel evolving lines. One generation of the complex life cycle with multiple social traits involved the growth of *M. xanthus* populations in nutrient-rich CTT liquid (with gentamycin) medium till O.D. _600 nm_ reached 0.3–0.4. These cultures were then spotted on starvation TPM hard agar (1.5% agar) plate for sporulation and fruiting body development. Next, only the spores (and not vegetative cells that failed to sporulate) were harvested by first incubating the *M. xanthus* populations at 50°C, after which they were transferred onto TPM hard agar (supplemented with 0.025% glucose) beds overlaid with *Escherichia coli* lawns in flasks for germination and predation. After incubation for 4 days on *E. coli* lawns, populations were harvested by adding 4 mL TPM buffer, shaking at 200 rpm, and either (0.04 mL) 1%, or (0.6 mL) 15% of harvested populations were transferred to fresh CTT liquid media with gentamycin (*M. xanthus* is naturally resistant to gentamycin whereas *E. coli* is sensitive to it). This selection regimen was repeated for 10 cycles.

To test how population bottlenecks affect multiple cooperative traits, we used *Myxococcus xanthus* as a model organism to study the effect of repeated population bottlenecks on four distinct social traits that were under selection during a lab evolution experiment (Fig 1). *M. xanthus* is a gram-negative soil bacterium with a complex life cycle. Multiple stages of *M. xanthus* life cycle were previously shown to be cooperative. These include sporulation [31,32], predation [33], germination [34], and growth [35]. We performed a lab evolution experiment under two distinct bottleneck regimens. During these experiments, all four cooperative traits mentioned above (growth, multicellular spore-filled fruiting body formation, germination, and predation) were under selection.

When bottlenecks are applied, the fate of cooperation depends not only on relatedness but also on costs and benefits, spatial structure, and trade-offs. According to Hamilton’s rule, rB > C (where r – coefficient of relatedness, B – benefit to the recipient, and C – cost to the actor), cooperation is favored when cost of cooperation is low, benefit is high and relatedness within the population is high (r close to 1) [36]. Several ecological, mechanistic, and demographic processes can influence this balance. Therefore, low costs make cooperation harder to exploit, whereas high costs increase the temptation to cheat [10,37,38]. Spatial structure can stabilize cooperation by limiting dispersal and directing benefits to relatives [39]. But local kin competition may counteract this benefit [40]. Trade-offs between traits can further constrain outcomes, favoring some cooperative traits while disfavoring others [41].

So, the stability of cooperative traits in microbial systems may be determined by a combination of mechanisms: high relatedness arising from bottlenecks and spatial structure, together with variation in the ratio of cost/benefit of cooperation and underlying trade-offs across different life history traits.

The four cooperative traits we examined in this study play distinct roles in the life cycle of *M. xanthus*. Sporulation [42–44] is a cooperative trait because it involves multicellular fruiting body formation, where some cells differentiate into spores while others lyse, contributing to the survival of kin. Germination efficiency is influenced by diffusible molecules [34], making it a social trait as it benefits from the presence of other germinating spores. Predation involves the secretion of extracellular enzymes and antibiotics that collectively kill and digest prey [45,46], thus making secreted antimicrobials and extracellularly digested prey a public good [47]. Finally, growth in proteinaceous media depends on the extracellular degradation of proteins via secreted enzymes [35], a strategy akin to cooperative invertase production in *Saccharomyces cerevisiae* [48].

Interactions between traits also matter [26,27,49–51]. Trade-offs between sporulation and germination are documented in microbes [52–54] and in plants, such as the seed size-number trade-off [55,56]. Given these patterns, we predicted that *M. xanthus* might also exhibit trade-offs between sporulation and germination. Since both are cooperative traits in *M. xanthus*, it remains unclear how selection acts on them under different population bottleneck regimes. Do stringent bottlenecks stabilize both traits equally, or do trade-offs lead to the selective enrichment of only one?

Building on the possibility of additional mechanisms influencing the stability of cooperation, we hypothesized that since in our study four cooperative traits of *M. xanthus* (sporulation, germination, predation, and growth) are under investigation, population bottlenecks may drive different evolutionary outcomes. One possibility is that, if trade-offs exist, bottlenecks selectively maintain one cooperative trait while the trade-offs cause another to decline. Alternatively, bottlenecks may favor a generalist strategy, where multiple traits persist at intermediate levels. A third possibility is that selection maintains polymorphic populations, where different genotypes specialize in distinct cooperative traits and coexist through frequency-dependent selection.

We demonstrate that stringent population bottlenecks selectively favor some cooperative traits, such as sporulation and growth, while relaxed bottlenecks promote others, such as predation and germination, supporting the trade-off hypothesis. We observed maintenance of higher diversity within the relaxed regimen relative to the stringent regimen. Computational model predicted the involvement of the cost of cooperation as an additional mechanism influencing the evolutionary stability of cooperative traits. This differential selection suggests that bottlenecks do not necessarily stabilize all cooperative traits equally but instead, in conjunction with other mechanisms, can drive distinct evolutionary trajectories. These findings offer broader insights into how population bottlenecks influence life history strategies and cooperative behaviors across biological systems, deepening our understanding of the evolutionary dynamics of social traits.

## Results

### Sporulation and growth are maintained or positively selected in stringent population bottleneck regimen

To understand the effect of population bottlenecks, we propagated *M. xanthus* populations in conditions in which four distinct social traits (growth, sporulation, germination, and predation) were under selection (Fig 1). During the lab evolution experiment, *M. xanthus* populations were propagated for 10 cycles, each consisting of growth, starvation-induced development and sporulation, and germination and predation on Escherichia *coli* lawns. Between each of the 10 transfer cycles, either 1% (stringent regimen) or 15% (relaxed regimen) of the *M. xanthus* population was transferred from the predation condition to the liquid growth condition (Fig 1). Importantly, even after a 1% population bottleneck, approximately 10^5^ cells were transferred between cycles. Thus, though the *M. xanthus* populations cannot be propagated when the bottleneck is more stringent than 1%, the 1% (i.e., stringent) bottleneck still allowed for some degree of competition and hence might not be affected strongly by genetic drift. After 10 cycles of transfer, evolved populations and clones were analyzed to test our hypothesis that not all but only a subset of cooperative traits will be positively selected under stringent bottleneck treatment.

First, we tested the effect of the evolutionary regimen on starvation-induced sporulation. Starving *M. xanthus* cells initiate an aggregative developmental process that culminates in the formation of multicellular fruiting bodies filled with spores. Previous studies have shown that population bottlenecks influence the evolution of aggregative behaviors in both *Dictyostelium *discoideum, *M. xanthus,* and fungi [15,16,57]. Consequently, we hypothesized that populations subjected to stringent population bottlenecks would exhibit improved aggregative development compared to populations subjected to relaxed population bottlenecks. To test this, we inoculated both the ancestral isolate and evolved populations onto starvation (TPM) agar plates. In such conditions, *M. xanthus* populations develop spore-filled multicellular fruiting bodies, allowing for visual monitoring. This qualitative assessment indicated that all replicate populations from the stringent population bottleneck regimen displayed enhanced fruiting body formation (S1a Fig) compared to populations from the relaxed regimen. Given the identical fruiting body phenotypes observed across replicate populations, we selected population D15 (from the 15% regimen) and population D1 (from the 1% regimen) as representative populations for further investigations.

Evolved populations often contain multiple genotypes, and interactions among these genotypes can influence the overall phenotype of the populations. Hence, we checked whether distinct clones isolated from one representative population exhibit similar fruiting body formation abilities as seen at the population level. For this, we randomly selected three clones from population D1 (from the 1% regimen) and three clones from population D15 (from the 15% regimen). A comparison of the developmental phenotypes among these clones revealed similarities to the phenotypes observed in their respective populations of origin (S1b Fig).

**Fig 2:**
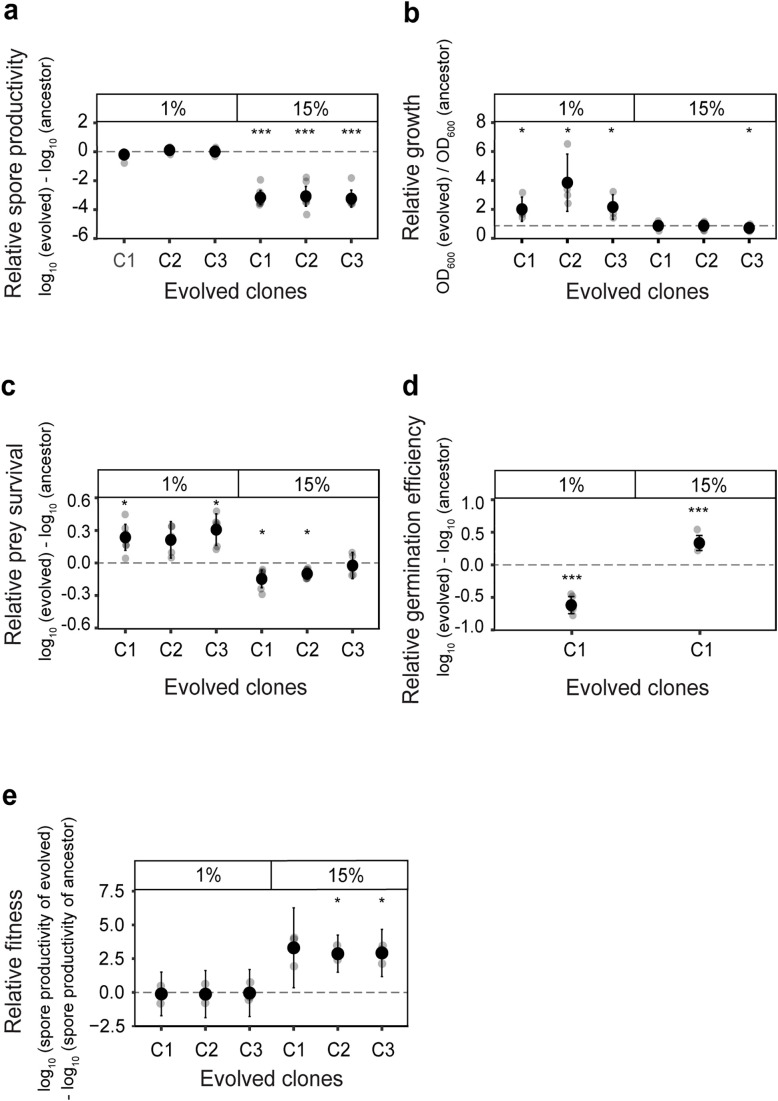
Stringent population bottleneck selects for faster growth and efficient sporulation, whereas relaxed population bottleneck selects for improved predation, germination efficiency, and higher competitive fitness across life cycles. Spore productivity, growth, predatory performance, and germination efficiency of three representative clones from 1% and 15% treatment were measured and plotted relative to the performance of their respective ancestors. (a) Data plotted is the spore productivity of evolved isolates relative to the ancestor (FDR corrected one-sample *t* test for differences relative to zero, *p* *** < 0.001, *n* = 7). (b) Clones from stringent population bottleneck treatment grow more in 48 hour relative to the ancestors. Data plotted is O.D. _600 nm_ of evolved isolates relative to the ancestral variant (FDR corrected one-sample t *t*est for differences relative to one, *p* * < 0.05, *n* = 3). (c) The growth of prey bacterium *Escherichia coli* was used as a measure of predation efficiency. Data shown is the growth (CFU/mL) of *E. coli* in the presence of evolved clones relative to the ancestors (FDR corrected one-sample *t* test for differences relative to zero, *p* * < 0.05, *n* = 7). (d) The percentage of spores germinated in 4 hour is used as the measure of germination efficiency. Data represent the germination efficiency of evolved clones relative to that of the ancestors (FDR corrected one-sample *t* test for differences relative to zero, *p* *** < 0.001, *n* = 6). (e) Clones from stringent (1%) and relaxed (15%) regimens were mixed with the ancestors in 1:1 proportion and the relative fitness of evolved clones against the common ancestor across two rounds of the life cycle was assessed. During this competition, clones from the 1% treatment were equally fit as their ancestors, whereas clones from the 15% regimen showed higher relative fitness (FDR corrected one-sample *t* test for signed square root *t*ransformed values for differences relative to zero, *p* * < 0.05, *n* = 3). The data used to produce all figures are provided in S1 Data folder.

Further quantitative analysis of the representative isolates revealed that, on average, clones from the 1% regimen exhibited a 3.14 log-fold higher spore production compared to the clones from the relaxed treatment (Fig 2a, independent-sample *t* test, *t* = 30.80, df = 4, *p*-value = 6.65 × 10^−^⁶). The higher productivity of clones from stringent treatment was not due to increased sporulation efficiency compared to the ancestors but rather a result of reduced spore productivity in the relaxed regimen clones (Fig 2a). These findings suggest that fruiting body development is selected against in the relaxed population bottleneck regimen, while repeated stringent population bottleneck events lead to the maintenance of sociality during sporulation.

Like sporulation, the growth of *M. xanthus* is also a density-dependent trait influenced by the availability of digested proteins and digestive enzymes [35], which act as public goods. In our evolution experiment, populations underwent growth in a liquid CTT medium, which contains Casitone-Tris buffer with potassium phosphate, before being transferred to the development plate for sporulation. Thus, we hypothesized that the population from the 1% regimen, which exhibited improved sporulation efficiency, would also demonstrate a superior growth phenotype. Indeed, clones derived from the 1% regimen exhibited a 3.26-fold higher productivity after 48h of growth in a liquid medium compared to clones from the 15% treatment (independent-sample *t* test between growth of clones from 1% regimen and 15% regimen, *t* = 3.14, df = 4, *p*-value = 0.0347). To complement growth data, we also measured protease secretion, which followed a similar trend: higher levels in 1% clones and lower in 15% clones (S2 Fig). Furthermore, the growth of the clones from the 1% regimen was significantly higher than that of their ancestors, while the growth of clones from the 15% regimen on average remained similar to that of their ancestors (Fig 2b). Taken together, our results indicate that stringent populations retained ancestral sporulation while improving growth, while relaxed populations performed worse than the ancestor in sporulation and remained comparable in growth.

During the evolution experiment, the transfer of *M. xanthus* spores onto *E. coli* lawns subjected the evolving populations to selection pressures for predation and germination on *E. coli*. Predation by *M. xanthus* involves the use of contact-dependent and independent molecules such as antibiotics and digestive enzymes to kill and digest prey cells [58–61]. These killing molecules, along with the extracellularly digested dead prey, contribute to the public good. To assess the predatory performance of evolved and ancestral *M. xanthus* isolates, *M. xanthus* cells were co-cultured with *E. coli* under conditions similar to the evolution experiment. These experiments revealed that the *M. xanthus* isolates that evolved in the 15% treatment exhibited higher predation efficiency compared to the isolates that evolved in the 1% regimen (Fig 2c, 1.04 log-fold lower CFU/mL of *E. coli* in the presence of *M. xanthus* from the 15% treatment compared to the clones from the 1% treatment, independent-sample *t* test, *t* = 7.74, df = 4, *p*-value = 0.0017). Furthermore, comparison between the ancestors and the evolved clones showed that relaxed population bottlenecks selected for increased predatory performance (on average 1.2-fold lower CFU/mL relative to the ancestors, one-sample *t* test, *p*-value < 0.0064^[FDR corrected]^ or did not affect the predatory performance (one out of three isolates tested)), while stringent population bottlenecks selected for decreased performance relative to the ancestors (on average 1.8-fold higher CFU/mL of *E. coli* in the presence of *M. xanthus* relative to the ancestors, one-sample *t* test, *p*-value < 0.0293^[FDR corrected]^) (Fig 2c).

Germination of *M. xanthus* spores is a density-dependent cooperative trait driven by diffusible public goods [34]. Due to the complexity and time-sensitive nature of measuring germination efficiency, we focused on studying one representative isolate from each treatment for this analysis. The estimation of germination efficiency in the evolved and ancestral isolates showed that the clones from the 15% regimen exhibited significantly higher germination rates (on average 100% spores germinated within 4 hour) compared to the ones from the 1% regimen (on average 0% spores germinated) (Fig 2d, two-sample *t* test, *t* = −28.09, df = 10, *p*-value = 7.603 × 10^−11^). These results further demonstrate that when multiple cooperative traits are under selection, only a few are positively selected or maintained in a stringent bottleneck regimen.

The differential performance of clones from the 1% and 15% regimens in growth, sporulation, germination, and predation could be attributed to the maintenance of cooperative traits or the evolution of variants that excel in these specific traits without relying on social interactions. To assess whether these traits involve social interactions, we utilized density dependence as a valuable tool. Cooperative behaviors often display density-dependent effects, with the benefits of expression of the trait increasing with higher population densities [62,63]. By examining how the traits respond to varying population densities, we can infer the presence of cooperation within the bacterial population. Thus, to demonstrate the cooperative nature of growth and sporulation in the clones from the 1% regimen, as well as the cooperative predation in the 15% regimen clones, we tested for the density-dependent performance of the evolved isolates for these respective traits. Due to logistical constraints, we were unable to measure the density dependence of germination efficiency of the evolved isolates (see Materials and methods). These experiments revealed strong positive density dependence for growth rate (ancestor: *R sq.* = 0.5917, *p*-value = 1.457 × 10^−7^; 15%: *R sq.* = 0.2178, *p-value* = 0.0095; 1%: *R sq.* = 0.2521, *p-value* = 0.0036) and sporulation efficiency (ancestor: *R sq.* = 0.2304, *p-value* = 0.0023; 15%: *R sq.* = −0.0022, *p-value* = 0.3927; 1%: *R sq.* = 0.5442, *p-value* = 1.318 × 10^−6^) for the 1% regimen clones. Similarly, the 15% regimen clones exhibited positive density dependence in predation efficiency (ancestor: *R sq.* = 0.9525, *p-value* < 2.2 × 10^−16^; 15%: *R sq.* = 0.9501, *p-value* < 2.2 × 10^−16^; 1%: *R sq.* = 0.8766, *p*-value* <* 2.2 × 10^−16^). Moreover, as expected, the ancestral variant displayed positive density dependence in each of the four traits (S3 Fig).

We observed that the evolved isolates from stringent and relaxed regimens evolved to perform better at two distinct sets of social traits. These findings suggested that the populations from the two regimens have evolved to survive using two distinct strategies. However, better or worse performance at individual traits may or may not result in a competitive advantage for the evolved variants against their ancestors when they compete with each other across a life cycle that involves all traits examined in our experiments. Hence, the evolved isolates were mixed with their ancestors in 1:1 proportion and propagated across the life cycles twice before their numbers were estimated to count competitive fitness (Fig 2e). Interestingly, we observed that the isolates from the 15% regimen outcompeted their ancestors repeatedly across replicates (3.02-log-fold higher productivity relative to ancestors, one-sample *t* test for signed square root transformed values, *p-value* < 0.0313^[FDR corrected]^). In contrast, the ones from the 1% regimen on average showed similar fitness to their ancestors (one-sample *t* test, *p-value* > 0.05). Together, isolates from the 15% regimen seem to have evolved higher degrees of adaptive fitness over their ancestors relative to the ones from the 1% treatment.

Taken together, distinct cooperative traits were either maintained or improved in the two regimens. Importantly, the traits enriched in one treatment were selected against in the other. Thus, demonstrating that the size of the population bottleneck can determine which cooperative traits are selected.

### Strains with lower spore productivity coexist alongside high-sporulating strains in the relaxed regimen

Low-sporulating strains in the relaxed bottleneck (15%) regimen may persist in the population by coexisting with high-sporulating variants and may benefit from the cooperative environment they create. To test this hypothesis, we analyzed heterogeneity and direct interactions among evolved clones. To do so, we isolated an additional eight clones for 15% treatment and nine for 1% treatment, resulting in a total of 11 clones for the 15% regimen and 12 for 1% regimen.

**Fig 3:**
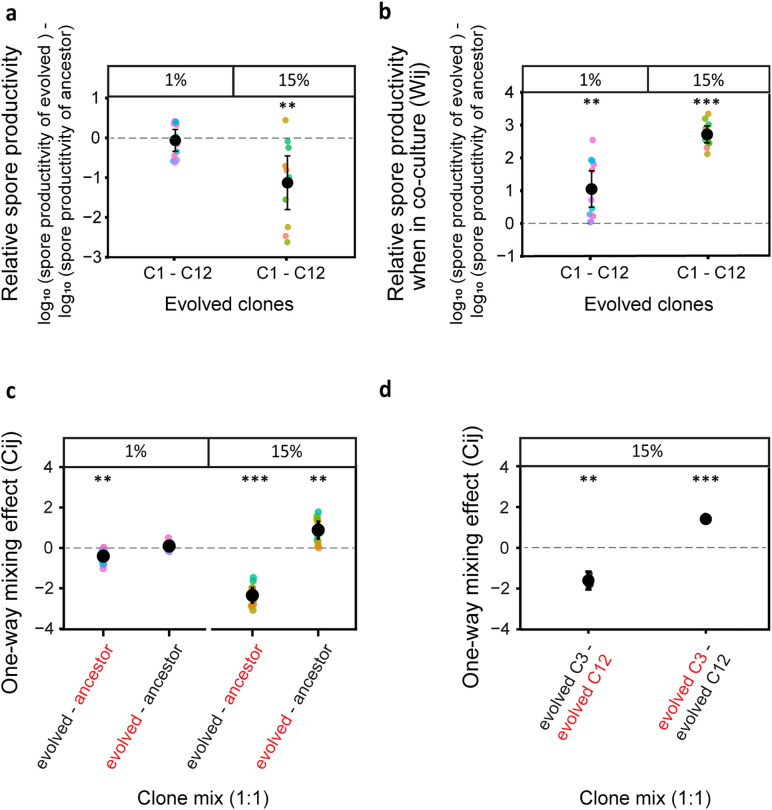
When additional clones from evolved populations were isolated and checked, 15% population show higher heterogeneity and consistently higher competitive fitness against the ancestor relative to 1% population. For an additional set of nine clones (12 clones in total) from 1% and 15% populations, the mono-culture spore productivities relative to ancestor and relative spore productivities when in co-culture with the ancestor are given. (a) Data plotted is the spore productivities for all isolated clones from 1% and 15% populations (total 12 each) relative to ancestor (FDR corrected one-sample *t* test for differences relative to zero, *p* ** < 0.01, *n* = 4) (b) The relative spore productivities (Wij) for all isolated clones from 1% and 15% when in competition against the ancestor is plotted (FDR corrected one-sample *t* test for differences relative to zero, **p < 0.01, ***p < 0.001, *n* = 4) (c) The Cij values indicate the one-way mixing effect on a clone (mentioned in red color in the X-axis) when in co-culture with other clone (mentioned in black color in the X-axis). Here, each of the 12 clones was independently mixed with ancestor at 1:1 ratio and the Cij values for their respective interaction effect are shown (FDR corrected one-sample *t* test for differences rela*t*ive to zero, **p < 0.01, ***p < 0.001 *n* = 4). (d) The Cij values given here are for the development competition between a good sporulating clone from 15% (C12) when mixed at 1:1 with a less sporulating clone from same population (C3) (FDR corrected one-sample *t* test for differences relative to zero, **p < 0.01, ***p < 0.001 *n* = 4). A negative Cij indicates reduced spore productivity for a clone relative to the expectation from its mono-culture productivity due to the presence of the other clone. A positive value of Cij indicates improved productivity for a clone relative to the mono-culture expectation due to the presence of the other clone. The data used to produce all figures are provided in S1 Data folder.

Sporulation assays revealed striking differences in within-regimen variability (Levene’s test for homogeneity of variance, F (1, 21) = 4.95, *p-value* = 0.0372). Clones from the stringent (1%) treatment showed uniformly high sporulation efficiencies comparable to the ancestor (Fig 3a; one-sample *t* test, df = 11, *t* = −0.528, *p-value* = 1 ^[FDR-corrected]^, standard deviation (SD) of 1% clones’ mean productivity = 0.43, interquartile range (IQR) = 0.84) while 15% clones had significantly lower average sporulation with markedly higher variance (Fig 3a; one-sample *t* test, *t* = −3.74, df = 10, *p-value* < 0.0077 ^[FDR corrected]^; SD = 1.00, IQR = 1.42). These results are consistent with the coexistence of both high and low sporulators in the relaxed bottleneck populations. Furthermore, competition assays between evolved clones and the ancestor highlighted contrasting interaction dynamics across the two regimens. Both 1% and 15% clones consistently outcompeted the ancestor during fruiting body development (Fig 3b). However, 1% clones had no significant benefit from the presence of the ancestor and often reduced the ancestor’s productivity, consistent with autonomous cooperative performance. In contrast, most 15% clones performed better in the presence of the ancestor (Fig 3c, positive one-way mixing effect (Cij), one-sample *t* test, df = 10, *t* = 4.68, *p*-value* = *0.0035), while simultaneously reducing the ancestor’s productivity (negative one-way mixing effect (Cij), one-sample *t* test, df = 10, *t* = −14.4, *p-value* = 2.14 × 10^−7^), a hallmark of exploitative interaction [64].

To test whether low-sporulating clones benefit from co-culture with high-sporulating clones (see S1 Table for respective sporulation efficiencies), we conducted pairwise interaction assays between C3 (a low-sporulating clone) and C12 (a high-sporulating clone) from the 15% treatment. When grown together, C3 exhibited a significant 1.4 log-fold increase in sporulation efficiency compared to when grown alone (Fig 3d, one-sample *t* test, *t* = 24.2, df = 3, *p-value* = 0.0003), indicating that it benefits from the presence of C12. At the same time, C12’s sporulation was reduced 1.6 log-fold in co-culture with C3, suggesting that C3 imposes a cost on C12 (Fig 3d, one-sample *t* test, *t* = −11.5, df = 3, *p-value* = 0.0028). This was confirmed by interaction indices, the one-way mixing effect (Cij) value for C12 was negative, reflecting reduced productivity due to C3, while the one-way mixing effect (Cij) value for C3 was positive, indicating improved performance in the presence of C12 (Fig 3d). Together, these data reveal a social exploitation dynamic in which low-sporulating strains like C3 can persist by benefiting from the cooperative investment of high-sporulating strains like C12, even while imposing a cost on them.

In summary, relaxed bottlenecks promote heterogeneity and facilitate the coexistence of exploiters and cooperators. In contrast, stringent bottlenecks select for homogeneous populations composed of robust cooperators. These findings underscore the role of population bottlenecks in shaping the evolution of cooperative traits, not only through direct selection but also by modulating the social context that enables exploitation.

### Mutations in regulatory genes are responsible for the changes

**Fig 4:**
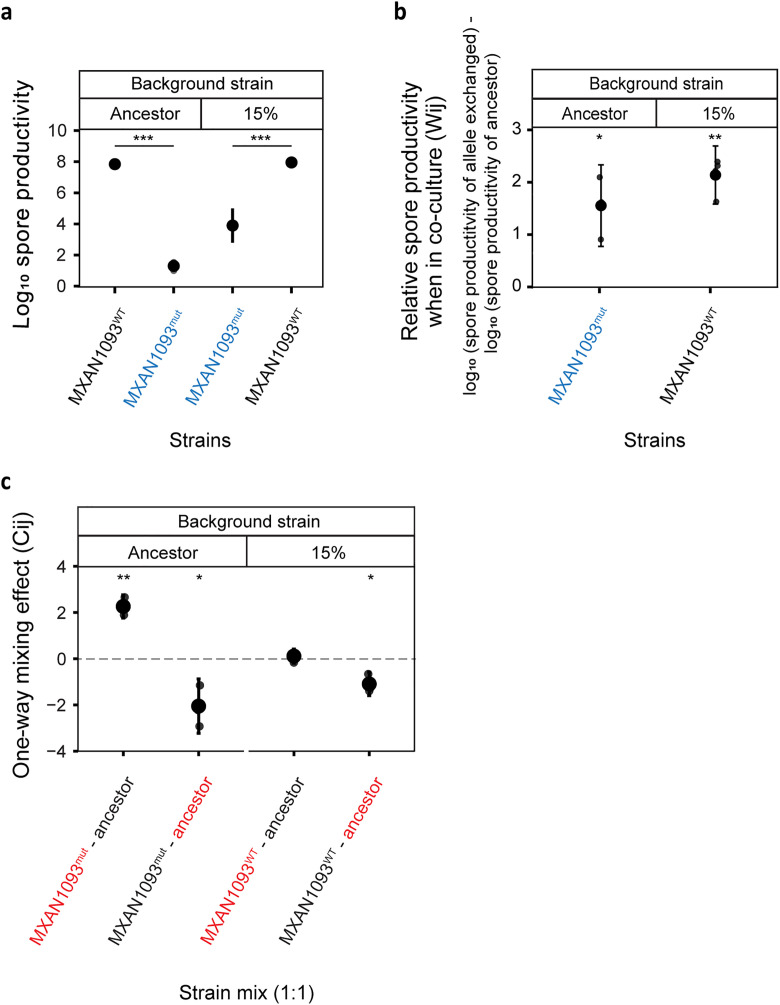
Point mutation in MXAN_1093 gene was responsible for the observed changes including higher competitive fitness in the evolved isolates from 15% treatment. Wild-type and mutant alleles of MXAN_1093 were genetically exchanged with their native alleles in evolved clone and ancestor backgrounds, respectively. Both final spore productivities and the competitive fitness against the focal ancestor GV2 are given. (a) Replacing the native allele in the ancestor background with the MXAN_1093 mutant allele resulted in a large reduction in absolute spore productivity (FDR corrected two-sample *t* test for differences between ancestor mean productivity and derived ancestor productivity, ***p < 0.001, *n* = 4). Replacing the derived allele in evolved 15% background with the ancestral allele resulted in an improvement of absolute spore productivity (FDR corrected two-sample *t* test for differences between evolved 15% mean productivity and derived 15% productivity post allelic exchange, *p* *** < 0.001, *n* = 4) (b) This figure shows the relative spore productivity (Wij) of allele-exchanged strains to ancestor GV2 when co-cultured (FDR corrected one-sample *t* test for differences relative to zero, *p < 0.05, **p < 0.01, *n* = 4) (c) The Cij values indicates the one-way mixing effect on the allele-exchanged strains by GV2 ancestor and vice versa when in co-culture with the ancestor. In the x-axis, the clone which induced the effect is given in red color and the clone which receives the effect is mentioned in black color (FDR corrected one-sample *t* test for differences relative to zero, **p < 0.01, *n* = 4). A negative Cij indicates reduced spore productivity for a clone relative to the expectation from its mono-culture productivity due to the presence of the other clone. A positive value of Cij indicates improved productivity for a clone relative to the mono-culture expectation due to the presence of the other clone. The data used to produce all figures are provided in S1 Data folder.

Three clones from 15%, as well as 1% treatment, were sequenced to identify the mutational changes relative to their respective ancestors. We found a small number of mutational differences between evolved isolates and their respective ancestors (S2 Table). Clones from the 15% regimen revealed that all three isolates acquired a missense mutation that was absent in their ancestors as well as among the clones from the 1% treatment. This mutation was responsible for the change in aspartic acid to asparagine at the 128th position of the DNA binding response regulator MXAN_1093 (S4 Fig). MXAN_1093 has been reported as one of the orphan response regulator proteins of the two-component system and was identified to involve in the initial hours of the fruiting body formation [65]. Thus, it was highly likely that the mutation identified in this gene in its signal receiver domain had a negative effect on its phosphorylation functionality and was responsible for the low spore productivity of isolates from the 15% regimen. To test this hypothesis, we replaced the ancestral variant of the allele with the evolved one in the ancestral isolates and replaced the derived variant of the allele with the ancestral variant in the evolved clones. The introduction of the derived allele in the ancestral variant resulted in a 6.55 log-fold reduction in the sporulation efficiency (Fig 4a, two-sample *t* test, df = 13, *t* = 40.34, *p-value* = 9.61 × 10^−15[FDR corrected]^) whereas exchanging evolved allele with the ancestral one in the derived strains resulted in the increase in sporulation efficiency by 4.06 log-fold (Fig 4a, two-sample *t* test, df = 14, *t* = −23.60, *p-value* = 2.25 × 10^−12[FDR corrected]^). Thus, the analysis of the reconstructed clones confirmed that the missense mutation in MXAN_1093 was indeed responsible for the reduction in the sporulation efficiency among derived isolates from 15% treatment.

To further evaluate whether the MXAN_1093 mutation influences not just sporulation efficiency but also social interaction dynamics, we conducted competition assays between allele-exchanged strains and the focal ancestor (GV2). These experiments revealed that the introduction of the MXAN_1093 mutant allele into the ancestral background resulted in a significant increase in competitive fitness during co-culture with the ancestor (Fig 4b, one-sample *t* test, *t* = 6.35, df = 3, *p-value* = 0.0158^[FDR corrected]^). However, introducing the wild-type MXAN_1093 allele into the evolved 15% background did not lead to a corresponding decrease in the evolved strain’s competitive fitness (Fig 4b, one-sample *t* test, *t* = 12.3, df = 3, *p-value* = 0.0023^[FDR corrected]^). This suggests that while the MXAN_1093 mutation contributes to the observed phenotype, additional mutations acquired during evolution likely also play a role in enhancing overall fitness.

One-way mixing effects (Cij) revealed asymmetric interactions between the allele-exchanged strains and the ancestor. When the MXAN_1093 mutant allele was introduced into the ancestral background, the resulting strain sporulated more efficiently in the presence of the wild-type ancestor than it did in mono-culture (Fig 4c, Cij > 0, one-sample *t* test, t = 14.3, df = 3, *p-value* = 0.0030^[FDR corrected]^), indicating that the sporulation efficiency of the ancestral background carrying the mutant allele increases in the presence of cooperators. Conversely, the wild-type ancestor showed reduced sporulation when co-cultured with the ancestral background carrying the mutant allele (Fig 4c, Cij < 0, one-sample *t* test, *t* = −5.56, df = 3, *p-value* = 0.0459^[FDR corrected]^), suggesting that the mutant allele imposes a cost on the ancestor. These findings mirror the interaction asymmetries seen among naturally evolved clones from the 15% treatment and reinforce the interpretation that the MXAN_1093 mutation promotes exploitative interactions while contributing to fitness gains.

Similarly, genomic analysis of the isolates from the 1% regimen revealed a frameshift mutation in a sigma 54-interacting transcriptional regulator MXAN_4899 which was absent in the ancestors as well as the derived clones from the 15% regimen (S5 Fig). Our attempts to exchange the alleles between ancestors and the derived clones were unsuccessful. However, previous studies of MXAN_4899 strongly support our hypothesis that the mutation in this gene was indeed responsible for the observed phenotype. MXAN_4899 was previously shown to be responsible for the regulation of secondary metabolites and, hence, the predatory performance of *M. xanthus* [66]. Since this protein in its native form helps in the production of secondary metabolites, it is understandable that a frameshift mutation (potential loss of function) might be responsible for the reduced predatory performance of the isolates from the 1% regimen. Why this frameshift mutation which results in accessibility to a new stop-codon and thus, presumably an altered protein structure might affect the growth of the isolates positively is not clear. We suspect that the cost saved by not producing the secondary metabolites involved in predation might be responsible for the increased growth of these mutants.

### Life-history traits of natural isolates are negatively correlated, indicating the presence of trade-offs in nature

**Fig 5:**
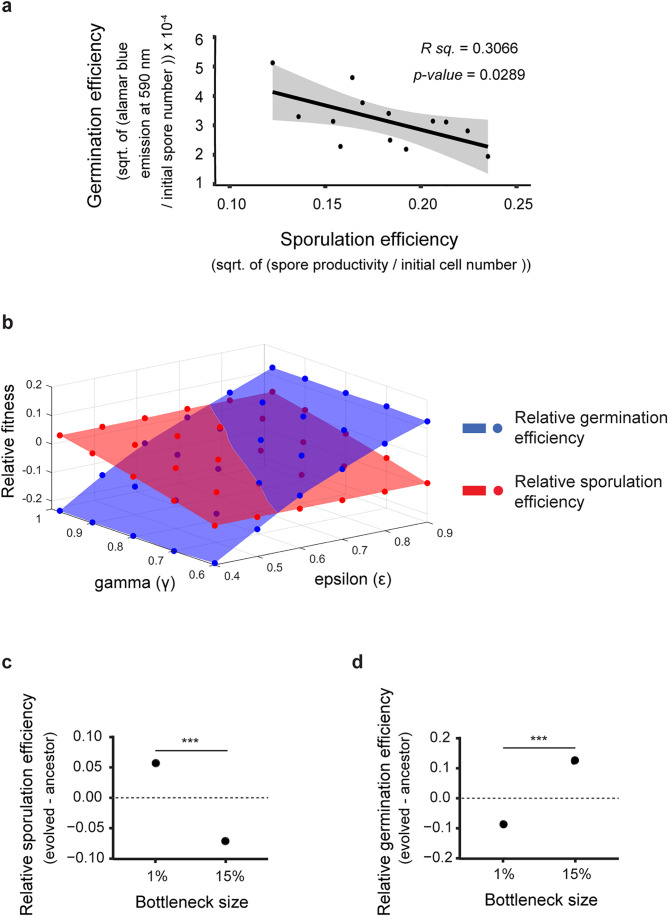
Computational model reveals that the cost of cooperation during sporulation (γ) and degree of privatization during germination (ε) can explain the evolution of life-history strategies as seen in the evolution experiment and in the natural isolates of *M. xanthus.* **(a)** Spore productivity of 13 randomly chosen soil-derived *M. xanthus* isolates was measured in conditions similar to the ones used during the evolution experiment and correlated with their germination efficiency. The line indicates fitted linear regression (*R. sq.* = 0.3,066, and *p* = 0.0289, *n* = 4). **(b, c,** and **d)** Spore productivity and germination efficiency with respect to ancestors were computed from a simulation model for a range of parameters – γ and ε (b) 3-D data plotted is the relative spore productivity and relative germination efficiency for parameter values ranging from 0.4 to 0.9 (ε) and 0.6 to 0.98 (γ) (c) A low cost of cooperation (γ close to 1) during sporulation resulted in improved sporulation efficiency in 1% and a high cost of cooperation (γ far from 1) resulted in the reduction of sporulation in 15% system (γ = 0.98 (1%) and γ = 0.65 (15%)) (d) A high privatization of public goods during germination (ε far from 1) resulted in reduction of germination efficiency in 1% and a low privatization during germination (ε close to 1) resulted in improved germination efficiency in 15% (ε = 0.5 (1%) and ε = 0.9 (15%)). The data used to produce all figures are provided in S1 Data folder.

Our hypothesis that trade-offs between different cooperative traits might prevent all cooperative traits from being enriched in response to population bottlenecks was based on the expectation that some cooperative traits would inherently conflict. We specifically predicted this for sporulation and germination, given that similar trade-offs have been documented in species where these traits are not social. For example, sporulation and germination trade-offs in *Bacillus subtilis* [52] and seed size-number trade-offs in plants [55]. To test whether the trade-off observed in our lab-evolved *M. xanthus* populations also occurs in natural populations, we examined the relationship between sporulation (i.e., development) and germination efficiency in wild isolates. We selected these two traits because they showed the highest divergence between populations evolved under 15% and 1% regimens (Fig 2a and 2d). For this analysis, we randomly selected 13 natural isolates of *M. xanthus*. Estimates of germination efficiency using Alamar dye and starvation-induced sporulation efficiency revealed a significant negative correlation between the two traits (Fig 5a, Pearson correlation test, *r* = –0.60, *t* = –2.51, df = 11, *p-value* = 0.0289). These findings suggest that the trade-off we observed between spore productivity and germination efficiency may be a general constraint on *M. xanthus* social evolution in nature that drives the evolution of life cycles of *M. xanthus* in natural populations.

### Population-wide computational modeling of the synthetic life cycle predicts the role of cost of cooperation and degree of privatization as the key players in deciding the outcomes

According to Hamilton’s rule, cooperation can stabilize when the coefficient of relatedness (r) and the benefit to the recipient (b) are high and the cost of cooperation for the actor (c) is low. Because the majority of the cells die during the formation of multicellular spore-filled fruiting bodies, the cost of cooperation during fruiting body formation is very high. As the population can withstand starvation and survive, the benefits associated with multicellular development and sporulation are also high. One well-established mechanism for stabilizing cooperation is the reduction of its associated costs [37,38]. Hence, we hypothesized that the evolution of fruiting body proficient variants in the 1% regimen must be associated with the reduced cost of cooperation, which might allow the evolution and maintenance of such traits. To test this hypothesis, we devised a mathematical model that resembled the selection regimen used during the evolution experiment. However, as opposed to the original lab setup life cycle, which had a predation stage as well, we removed this specific stage from the model since we were investigating the problem based on the germination-development trade-off. Additionally, the model also incorporated appropriate spatial structures for these two cooperative traits as it is a tertiary stabilizing mechanism for cooperation. As in the lab evolution experiment, germination stage was modeled as a well-mixed-system while multicellular development leading to sporulation was modeled with a surface associated spatial structure to ensure assorted local interactions.

The model, designed on a population-wide scale, mimicked the synthetic life cycle of *M. xanthus* as attempted in the lab evolution experiment (See Fig 1). Here, the simulation began with an initial population size of 1,000,000 cells, each of which was assigned ancestral sporulation and germination efficiency values (S7a,b Fig). These values were allowed to change over generations with a constraint that the sporulation and germination efficiencies were negatively correlated. This is because of a statistically significant negative correlation between these two traits among the natural isolates of *M. xanthus* (Fig 5a). This change over generations represented the evolution of trait values.

In the model (S3 Table), during each life cycle, individuals were allowed to first sporulate while interacting with individuals in the environment that had either higher or lower sporulation efficiency. Interactions between individuals were modeled using the game theory approach using a pay-off matrix as described in S4 Table, wherein γ (gamma) represented the cost of cooperation. Next, sporulated cells were allowed to germinate. The germinating individuals germinated at the efficiency that is the average efficiency of the population. This was because the germination of *M. xanthus* spores has been known to depend on diffusible public good molecules [6,53] and during the evolution experiment, the fruiting bodies were mixed post-harvest, such that there is minimal effect on cooperation due to the spatial structure. This approach allowed us to model the degree of privatization of public goods represented as ε (epsilon). Thus, our approach to calculate germination efficiency allowed us to modulate the degree of public good privatization and hence effective cost of cooperation during germination as well.

We implemented two life cycle regimens: one with a bottleneck at a transfer size of random 1%, and another where a random 15% of the population was advanced to the next stage (S3 Table). As we explored a broad range of parameter spaces for γ and ε, the lab-evolved and computationally simulated populations exhibited similar trends based on the values of γ and ε (see Fig 5c and 5d). A high degree of privatization of public goods during germination (ε close to 0.5), along with a low cost of cooperation during development (γ close to 1), resulted in the selection of variants that were proficient at sporulation but had poor germination efficiency (Figs 5b and S6). These results are similar to the ones observed in the 1% regimen. In contrast, a liberal sharing of public goods during germination (ε close to 1), along with a high cost of cooperation during development (γ far from 1), selected for variants that were poor sporulators but efficient germinators (Figs 5b and S6). These results were similar to the ones observed in the 15% regimen.

Interestingly, the size of the population bottleneck during the simulation had no impact on the outcomes of the model. However, at distinct sets of values of γ and ε both 1% and 15% bottleneck sizes recapitulated their respective trade-off outcomes. Thus, the modeling strategy demonstrated that an important way the strategies as seen in the 1% regimen can evolve could be because of mutations that reduce the overall cost of cooperation during germination and sporulation. In contrast, the high cost of cooperation during germination and sporulation selected for strategies seen in the evolved strains from the 15% regimen. Moreover, after 10 cycles of iterations, the model was able to effectively replicate the evolution of traits such as fruiting body development and germination, mirroring what was observed in lab-evolved populations, including the flip of trade-offs (Fig 5c and 5d).

The distribution of cells for their ability to sporulate or germinate also changed widely from the original population at the start of the cycle and between the two regimens. Parameters that led to the outcomes as seen in the 1% regimen showed a skewed distribution for both sporulation and germination towards higher productivity but still harbor heterogeneity (S7c,d Fig). For parameters that result in the outcomes seen in the 15% treatment, the distribution was wider; the observed heterogeneity in the 15% treatment is a result of increased genetic variation and clonal interference, which are facilitated by the larger population bottleneck (S7e,f Fig). Finally, we tested whether the outcomes from the model were stable over long evolutionary time. For this, we estimated the proportions of the cheaters and cooperators over time. This analysis revealed that the outcomes from the model were stable over many additional generations. This was true for the both parameter spaces that resulted in the respective outcomes as seen in the 1% regime and the 15% regime (S7g,h Fig). Hence, though our model does not demonstrate why certain strategies are selected in the 1% treatment and others in the 15% treatment, it does reveal that mutations that reduce the cost of cooperation might be important for the evolution of strategies seen among clones evolved in 1% treatment.

In our experiment, sporulation was an essential trait. Only individuals that sporulate were propagated further during the evolution experiment. We predict that the cells that have either similar or higher sporulation efficiency than the ancestors would get fixed in the population. In the stringent (1%) treatment, higher relatedness among individuals limited the scope for cooperator–cheater interactions. Under such conditions, cooperation can already be stable because exploitation opportunities are rare. Our modeling further suggests that cooperation was reinforced by reductions in the cost of sporulation, consistent with Hamilton’s rule that stability depends on the joint effects of relatedness, benefits, and costs.

The reduced cost of cooperation under the 1% bottleneck likely stems from two complementary processes. Repeated stringent bottlenecks purge exploiters, yielding genetically homogeneous populations in which all individuals contribute to sporulation, thereby lowering its effective cost. In addition, our data and model suggest that mutations such as the MXAN_4899 frameshift, which likely down-regulate energetically costly secondary metabolite production, might further decrease the metabolic burden of cooperation. Together, the absence of cheaters and reduced physiological investment explain why cooperation remains stable under the 1% regimen. This combination explains why sporulation was strongly maintained in 1% populations. In contrast, the relaxed (15%) treatment maintained greater diversity and a mixture of social strategies. Here, sporulators and nonsporulators could both persist, supported by frequency-dependent interactions that prevented any single strategy from fixing.

Overall, our model highlights that bottleneck sizes alone cannot explain the evolution of cooperative traits in *M. xanthus*, and additional mechanisms like the cost of cooperation and the degree of public good privatization are stronger influences. While stringent bottlenecks may increase relatedness, our findings suggest that cooperation is more likely to be maintained when the cost of cooperation is reduced, as seen in the 1% regimen. This suggests that mutations reducing the cost of cooperation are critical for the additional stability of cooperative traits under stringent bottlenecks.

Conversely, when the cost of cooperation remains high and public goods are more freely shared, as in the 15% regimen, selection favors variants that optimize germination efficiency at the expense of sporulation. However, our model does not fully explain why specific strategies emerged in each regimen, indicating that additional ecological and genetic factors—such as clonal interference, mutation accumulation, or population structure—may also play a role. Future work incorporating these complexities will be necessary to refine our understanding of how population bottlenecks shape the evolution of cooperation.

## Discussion

Explaining the evolution and maintenance of cooperative interactions poses a challenge. Previous studies have shown that population bottlenecks can stabilize cooperation, primarily by increasing kinship among individuals [14–16,67]. However, most of these studies have focused on examining a single cooperative trait [14–16]. Considering that many microbes possess multiple social traits [24–26], we sought to investigate whether stringent population bottlenecks would select for all cooperative traits or not. The results presented demonstrate that when stringent bottlenecks are repeatedly applied, only a select few cooperative traits are favored. Interestingly, the cooperative traits of sporulation and growth, which are either maintained or positively selected under stringent conditions, do not exhibit improvement in the relaxed regimen. Conversely, in the relaxed condition, germination and predation are favored. Notably, relaxed bottlenecks generated heterogeneous populations in which high-sporulating cooperators coexisted with low-sporulating exploiters, leading to higher overall fitness and diverse social strategies. In contrast, stringent bottlenecks produced more homogeneous populations of cooperators, consistent with high relatedness in both laboratory-evolved and simulated populations. These trade-offs between cooperative traits were also evident in natural isolates, reinforcing the ecological relevance of our findings.

In line with numerous other microbial species, *M. xanthus* exhibits the ability to express multiple social traits, such as swarming, growth, sporulation, germination, and predation. These traits are predominantly driven by diffusible substances [34,35,68] and, in some cases, additional contact-dependent interactions [32,60]. Each of these social traits can be studied under distinct environmental conditions, enabling us to replicate the life cycle of *M. xanthus* in a laboratory evolution experiment. In this experiment, *M. xanthus* populations were grown in a nutrient-rich environment, transferred to starvation agar for development, and then transferred to growing prey lawns for germination and predation. Thus, sporulation, germination, predation, and growth were all subjected to selection.

Our criteria for defining a trait as social were based on whether *M. xanthus* populations show density-dependent performance for the respective trait. Such density dependence is generally mediated by diffusible molecules that typically provide higher benefits with increasing concentration. For example, siderophores production for iron chelation in *Pseudomonas sp*, extracellular digestion of sucrose in yeast, quorum sensing in bacteria, etc [6–8]. Two of the four traits, i.e., sporulation and germination have been reported to be density-dependent in previous studies [32,34]. Moreover, as in the other examples of microbial social traits mentioned above, diffusible molecules play an important role during sporulation and germination of *M. xanthus* [32,34]. Amongst the remaining two traits, this study is the first one to demonstrate predation as a density-dependent trait. Two aspects of *M. xanthus* predation suggest that density-dependent predatory performance is expected. First, the use of diffusible prey-killing molecules [33,58,59,61] by *M. xanthus* can serve as *“public goods”*. Second, extracellular digestion of prey involves the secretion of lytic enzymes and thus the enzymes and digested prey can serve as *“public goods”*. Finally, growth has been shown to be density-dependent only in certain environmental conditions [63].

Furthermore, during the evolution experiment, *M. xanthus* populations were propagated in distinct media types that allowed us to replicate the life cycle of *M. xanthus* under controlled conditions. Some of the media conditions used here were structured (e.g., development, germination, and predation agar medium), whereas for growth, the medium used was liquid. These media conditions were chosen to systematically study the four cooperative traits [69]. It is well established that spatial structure affects the ecology and evolution of cooperative traits. Thus, it is possible that if the experiments were repeated under different spatial conditions, the outcomes might have varied. A structured environment can stabilize cooperation by limiting dispersal, reducing diffusion of public goods, and promoting assortment among relatives [39]. However, local competition within kin groups can counteract these benefits [40]. Relatedness in such systems is shaped by local interaction density [70]. Thus, in addition to differences in relatedness between relaxed and stringent regimens, the scale of competition varied across traits due to spatial structure. Yet sporulation was maintained in stringent clones despite the expectation of heightened local competition. Our model suggests that reduced costs of cooperation provided an additional stabilizing mechanism under these high relatedness, high-competition conditions. Therefore, although variations in spatial structure could influence the specific evolutionary trajectories observed, it is likely that bottleneck effects together with other stabilizing mechanisms would still be nonuniform across different cooperative traits, with some being positively selected while others were selected against due to trade-offs.

While all four social traits were under selection, growth and sporulation experienced stronger selection pressures compared to germination and predation. This was because, after being spotted on starvation agar, all cells that failed to transition into spores were eliminated before transfer to the prey lawn. Hence, the ability to sporulate, either in isolation or in the presence of good sporulators, was crucial for the survival of individuals. Once inoculated on the prey lawn, spore population could afford to germinate slowly and possess less efficient predation mechanisms since, after 4 days of coincubation, the cultures were transferred to a nutrient-rich liquid medium. Superior growth in the liquid medium ensured that individuals were transferred to the starvation agar plate for the next round of the life cycle. Therefore, growth and sporulation had a greater impact on evolutionary success than germination or predation. This does not imply that sporulation or growth are “essential” in a binary sense, but rather that the ecology of the experimental life cycle imposes stronger filtering on some traits than others.

Population bottlenecks strongly influence adaptation. Under such conditions, the first beneficial mutations that arise by chance face relatively weaker clonal competition and are more likely to fixate, especially when selection pressure is high [28,29,71,72]. In the evolution experiment conducted in this study, populations underwent stringent bottlenecks just before being transferred to a growth medium. Thus, any variant that exhibited superior fitness during the growth phase in the liquid medium would increase in frequency and survive if it also possessed the ability to sporulate. Our results strongly support this evolutionary dynamic as the driving force behind the evolution of variants that perform better during liquid growth and sporulation under stringent treatment compared to the relaxed treatment. As expected from this hypothesis, the variant enriched in our experiment demonstrated both strong growth capabilities and maintained similar levels of sporulation efficiency as its ancestor, resulting in its fixation. Furthermore, given the trade-off between sporulation and germination, the enrichment of efficient sporulators automatically resulted in the decline of germination efficiency among evolved populations in the 1% treatment.

In contrast, under relaxed bottleneck treatment, higher levels of genetic diversity and competition enabled the selection of the fittest variants. Such outcomes are also consistent with a mutation-supply argument. In larger populations, such as those under the 15% regimen, higher effective population size increases the rate at which new mutations arise, making it likely that both cooperative and noncooperative variants appear and coexist. Given the trade-off between sporulation and germination, the emergence of low-sporulating variants would automatically favor improved germination efficiency, even without invoking social selection. However, our results suggest that this genetic diversity did not arise or persist through mutation supply alone. The observed frequency-dependent fitness effects, where low-sporulating isolates gained in the presence of cooperators but imposed costs on them, indicate that social interactions further shaped these outcomes. Thus, both mutation supply and social interactions together structured the observed diversity in the relaxed bottleneck regimen.

Our results demonstrate that efficient sporulation was not the optimal competitive strategy. Instead, the ability to sporulate better in the presence of an efficient sporulator was favored. This finding aligns with previous research, suggesting that variants capable of exploiting cooperative traits outcompete individuals investing resources in those traits. For these exploitative variants to succeed, it was not necessary for them to increase in frequency during growth. Instead, their ability to exploit sporulating strains during starvation was sufficient for their transfer to the next environment, such as *E. coli* lawns for germination and predation. Thus, relaxed bottlenecks maintained both cheaters and high-sporulating cooperators, ensuring population survival during starvation. Similar coexistence is reported in other cooperative systems, such as *Neurospora* and *Dictyostelium*, where frequency-dependent selection maintains a balance of social strategies [57,73]. Moreover, trade-offs between sporulation and germination, seen both in our experiments and in natural isolates, further enhanced the advantage of developmental cheaters that also germinated efficiently.

Taken together, our study demonstrates that when multiple social traits are under selection, stringent population bottlenecks tend to select for the maintenance of some cooperative traits. Because of trade-offs between sporulation and germination/predation, selection for sporulation in the stringent bottleneck condition led to a decline in the performance of nonessential social traits. In contrast, relaxed population bottlenecks selected for reduced performance and the evolution of exploitation in essential cooperative traits while favoring increased efficiency in nonessential social traits. Thus, we provide evidence that stringent bottlenecks do not always lead to the maintenance of cooperation and can sometimes result in reduced cooperativity. Considering that most microbial species exhibit multiple social traits [23,74] these findings hold broad relevance.

Our results indicate that while stringent bottlenecks increase relatedness, their effects on cooperation are not solely driven by genetic drift. Although extreme bottlenecks can create variation in social composition [75], the relatively large population sizes in our 1% treatment (~10⁵ cells per transfer) suggest selection, rather than stochasticity, shaped trait evolution. Parallel trends across independent replicates further support this, and our model results reinforce that cooperation is maintained only when its costs are sufficiently reduced. Thus, selection, not bottleneck size alone, determines the fate of cooperative traits, with trade-offs and cooperation costs playing key roles.

Additionally, the impact of bottlenecks on cooperation is likely nonlinear, varying by trait. Prior studies suggest cooperation is optimized at intermediate bottlenecks, where populations remain large enough to sustain cooperative interactions but small enough to purge exploiters [76]. Our results do not contradict the hypothesis that different traits can have different optimal bottleneck intensities. Furthermore, the population restoration phase (liquid growth) ensured that population size was not limiting, yet distinct evolutionary outcomes still emerged. These findings highlight the need to consider both bottleneck size and frequency when evaluating the evolution of microbial cooperation.

Complex life cycles are common, ranging from aggregative multicellularity seen in *M. xanthus* and *D. discodieum* to life cycles involving two developmental stages, such as larval development followed by metamorphosis or terminal development [77,78]. Our observations suggest that population bottlenecks within such life cycles influence the overall evolution of life history strategies [79]. Therefore, based on our findings, future studies could explore how population bottlenecks drive the evolution of life cycles in diverse microbial and multicellular systems.

## Materials and methods

### Strains and culture conditions

*Myxococcus xanthus* strains used in this study were GJV1 and GJV2 (a rifampicin-resistant version of GV1), obtained from Gregory Velicer, ETH Zurich [80]. The *E. coli* strain used in the study was the laboratory strain MG1655 [81]. All *M. xanthus* strains were stored in CTT liquid medium (composed of Casitone-Tris buffer with potassium phosphate) [69] containing 20% glycerol at −80°C, while *E. coli* was stored in LB medium with 20% glycerol at −80°C.

To obtain *M. xanthus* cultures for assays, the strains were inoculated from frozen stocks onto CTT hard agar (1.5% agar, 1% casitone, pH 7.6) plates and incubated at 32°C for 3 days, during which *M. xanthus* swarms appeared on the plates. Edges of the 3-day-old swarms were inoculated into 8 mL of CTT liquid medium (1% casitone, pH 7.6) in 50 mL conical flasks and incubated at 32°C with constant shaking at 200 rpm until they reached the desired optical density (O.D. _600 nm_, 0.2–0.8). A standard curve was used to convert O.D. _600 nm_ measurements to calculate resuspension volumes and adjust the cell densities (cells/mL). To demonstrate the effect of *M. xanthus* cell density on growth, predation, and sporulation, overnight cultures were serially diluted to different cell densities after an initial adjustment to a higher density of 5 × 10¹⁰ cells/mL and followed by respective assays (see later sections).

To obtain spores, cultures grown in CTT liquid medium were centrifuged at 5,000 rpm for 20 min at 25°C, and cell pellets were resuspended to a density of 5 × 10⁹ cells/mL in TPM buffer (pH 7.6). Aliquots of 100 µL (unless otherwise specified) were spotted onto TPM hard agar (1.5% agar, pH 7.6) plates and incubated at 32°C for 3 days. Fruiting bodies were harvested in 1 mL of ddH₂O using a sterile scalpel, then heated at 50°C for 2 hours and sonicated for 20 s (Amplitude: 25, Pulse: 10 s ON, 10 s OFF, 10 s ON) using a Q700 sonicator (Qsonica) with a 24-tip horn (part #4579). To demonstrate the effect of spore density on germination, the spores were initially adjusted to 5 × 10⁷ spores/mL and then diluted to different starting densities (refer to the germination assay section using Alamar dye for more information).

*E. coli* cultures were initiated by streaking glycerol stocks onto LB agar medium, followed by incubation at 32°C overnight. A single colony from the LB agar plate was inoculated into 8 mL of Luria Broth in a 50 mL conical flask and grown overnight until the O.D. _600 nm_ reached 1–1.2. The culture was then washed once and further adjusted to 0.1 O.D. _600 nm_ using TPM buffer for use in all predation assays.

### Isolation of natural *M. xanthus* isolates

To obtain natural isolates of *M. xanthus*, soil samples were collected from various locations on the Indian Institute of Science (IISc) campus, Bengaluru, India. Sterile 10-mL syringes with the tops cut off were used to collect the soil. After removing ~2 mm of the topsoil from inside the syringe using a sterile scalpel, the remaining soil was crushed and spread on selective medium [82] (TPM hard agar (1.5% agar) with 0.5% casitone, vancomycin (10 µg/mL) (Sigma Aldrich, V2002, CAS number 1404-93-9), nystatin (1 U/mL) (Sigma Aldrich, N4014 50MG, CAS number 1400-6-19), cycloheximide (50 µg/mL) (Sigma Aldrich, 01810-5G, CAS number 66-81-9) and crystal violet (10 µg/mL) (Sigma Aldrich, C0775, CAS number 548-62-9).

The soil-covered plates were incubated at 32°C for over 2 weeks, until fruiting bodies appeared. Thirteen different fruiting bodies from distinct locations were randomly collected and transferred to separate microcentrifuge tubes containing 1 mL of sterile ddH₂O using a toothpick. These samples were incubated at 50°C for 2 hours to kill any vegetative cells and enrich thermoresistant spores. The samples were then sonicated for 20 s (Amplitude: 25, Pulse: 10 s ON, 10 s OFF, 10 s ON) to release the spores from the fruiting bodies, followed by dilution and plating on CTT-soft agar (0.5% agar) media.

The spores germinated on CTT-soft agar plates over 4–5 days were collected and used for CTT-liquid inoculation, with each colony-forming unit on the soft agar plate representing a spore. Individual genotypes were designated as X.Y, where X denotes the fruiting body from which the spore originated, and Y represents the distinct spore number from that fruiting body. Cultures were incubated at 32°C with shaking at 200 rpm until they reached 0.2–0.8 (O.D. _600 nm_), after which they were frozen at −80°C in 20% glycerol.

### Isolation of *M. xanthus* clones from evolved population of *M. xanthus*

To better understand the within-population dynamics, we isolated clones from evolved populations as follows: The evolved population glycerol stocks were serially diluted and plated on CTT soft agar (0.5% agar) in 90 mm Petri dishes. After 4–5 days of incubation at 32°C, individual colonies (three from both the ancestor and evolved populations) were picked and used to inoculate 8 mL of CTT liquid medium in a 50 mL conical flask. Colonies were selected from plates that showed more dispersed and diverse colonies. When the cultures reached an O.D. _600 nm_ of 0.2–0.8, they were stored in 20% glycerol and frozen at −80°C. The three clones from each treatment were designated as C1, C2, and C3.

### Experimental evolution

To replicate the different life stages of the *M. xanthus* life cycle, three distinct growth conditions were used (S1 Fig). For vegetative growth, 50 mL conical flasks with 8 mL CTT liquid medium (1% casitone, pH 7.6) were used. For development and sporulation, TPM hard agar (1.5% agar) beds were made by pouring 10 mL of TPM hard agar into 60 mm Petri dishes. For the germination and predation phases, *M. xanthus* spores were co-inoculated with *E. coli* on 10 mL TPM hard agar medium (1.5% agar, 0.025% glucose) in 50 mL conical flasks. Since glucose was the only carbon source, *M. xanthus* relied on its predatory abilities to grow by using the growing population of *E. coli* as its sole nutrient source. Four replicate lines were propagated for each bottleneck condition, with four distinct GJV1 *M. xanthus* colonies used as ancestors for each replicate population.

To initiate the evolution experiment, individual *M. xanthus* colonies were inoculated in 8 mL of CTT liquid medium in 50 mL conical flasks for each replicate population. The cultures were grown until the O.D. _600 nm_ reached 0.3–0.4, and the cell density was adjusted to 5 × 10⁹ cells/mL using TPM buffer after centrifuging at 5,000 rpm for 20 min at 25°C. The density was adjusted to ensure that the overall effects we see were not because of the differences in the population sizes throughout the evolution experiment. Two hundred microliters of the adjusted culture were spotted on TPM hard agar plates and incubated for 3 days at 32°C. This incubation period was sufficient for *M. xanthus* cells to aggregate and form spore-filled multicellular fruiting bodies. After 3 days, the TPM plates were incubated at 50°C for 2 hours to kill vegetative cells while preserving the spores. The surviving spores were harvested by scraping the agar surface with a sterile scalpel and resuspended in 1 mL TPM buffer (pH 7.6).

The resuspended spores and an *E. coli* suspension (0.1 O.D. _600 nm_, 50 µL) were co-inoculated on 10 mL TPM hard agar (1.5% agar, 0.025% glucose) in 50 mL conical flasks and spread using 5–7 glass beads by shaking for 5 min. The flasks were incubated for 4 days at 32°C. After incubation, the cultures were harvested using glass beads, with 4 mL of TPM buffer added, followed by shaking at 200 rpm for 30 min. Depending on the population bottleneck size, either 40 µL or 600 µL of the culture was transferred to 8 mL CTT (with 50 µg/mL gentamycin) in 50 mL conical flasks and incubated at 200 rpm at 32°C until the cultures reached an O.D. _600 nm_ of 0.3–0.4.

We used a 40 µL bottleneck (1%) as trial experiments revealed that bottlenecks more stringent than this resulted in the death of the *M. xanthus* populations. A 600 µL bottleneck (15%) was used to ensure a significantly relaxed bottleneck, higher than most *M. xanthus* experimental evolution studies, which typically use bottlenecks closer to 10%. *M. xanthus* is naturally resistant to gentamycin, while the *E. coli* strain used was gentamycin-sensitive, allowing only *M. xanthus* to grow. Thus, the population size of *M. xanthus* was equalized across treatments at the end of each life cycle. CTT-grown cultures were centrifuged for 20 min at 5,000 rpm at 25°C, adjusted to a density of 5 × 10⁹ cells/mL using TPM buffer, and 200 µL was spotted on TPM hard agar plates to initiate the next cycle. The experiment was performed for 10 cycles. After every alternate cycle, glycerol stocks (20% glycerol) were made after growth in CTT liquid medium (with gentamycin 50 µg/mL) and before the next life cycle round, then stored at −80°C for analysis.

### Development assay

Aliquots from glycerol stocks, for both ancestor and evolved clones, were spotted on CTT hard agar (1.5% agar) plates. After 3 days of incubation at 32°C, the swarm edges were used to inoculate 8 mL of CTT liquid medium in 50 mL conical flasks. For evolved populations, cultures were obtained by inoculating a small aliquot of the freezer stock directly into CTT liquid medium. These cultures were incubated at 32°C with shaking at 200 rpm in 50 mL conical flasks until the O.D. _600 nm_ reached 0.3–0.4. The cultures were then centrifuged at 5,000 rpm for 20 min at 25°C, and the cell density was adjusted to 5 × 10⁹ cells/mL using TPM buffer. Aliquots of 100 µL were spotted onto TPM hard agar (1.5% agar) plates for standard development assays.

For qualitative analysis of the developmental proficiency of the evolved populations, plates were imaged after 3 days of incubation at 32°C. For quantitative analysis, after 3 days of incubation at 32°C, the plates were baked at 50°C for 2 hours. Spores were then scraped off the plates using a sterile scalpel, resuspended in 1 mL ddH₂O, and sonicated (Amplitude: 25, Pulse: 10 s ON, 10 s OFF, 10 s ON). Sonicated spore suspensions were serially diluted, and 100 µL of 10-fold dilutions were plated onto CTT soft agar. The plates were incubated at 32°C for 7 days, after which colonies were counted.

### Predation assay

Aliquots from glycerol stocks, for both ancestor and evolved clones, were spotted on CTT hard agar plates. After 3 days of incubation at 32°C, the swarm edges were used to inoculate 8 mL of CTT liquid medium in 50 mL conical flasks. The *M. xanthus* cultures in CTT liquid medium were incubated at 32°C with shaking at 200 rpm until they reached an O.D. _600 nm_ of 0.2–0.8. The *M. xanthus* cells were then centrifuged (5,000 rpm for 20 min at 25°C) and adjusted to 5 × 10⁵ cells/mL using TPM buffer.

We measured the growth of *E. coli* in the presence of *M. xanthus* as a measure of predatory performance. To do so, *E. coli* cultures were revived from glycerol stock on LB agar plates. A single colony was inoculated into 8 mL of liquid LB in 50 mL conical flasks for overnight growth. The cultures were then washed in TPM buffer and adjusted to an O.D. _600 nm_ of 0.1.

All predation assays were performed on 10 mL TPM hard agar (1.5% agar) beds supplemented with 0.025% glucose in 50 mL conical flasks, with 5–7 sterile glass beads on the surface of the agar bed. A 50 µL aliquot of density-adjusted *E. coli* culture (O.D. _600 nm_ 0.1) and 50 µL of density-adjusted *M. xanthus* (5 × 10⁵ cells/mL) were co-inoculated and spread on the agar bed with sterile glass beads. As a control, mono-cultures of *E. coli* were inoculated to estimate its growth in the absence of *M. xanthus*. For the control, 50 µL of density-adjusted *E. coli* culture (O.D. _600 nm_ 0.1) was inoculated with 50 µL of TPM buffer on agar beds and spread using 5–7 glass beads. Cultures were incubated at 32°C for 3 days.

Following the incubation period, 4 mL of TPM buffer was added to the conical flasks, and the culture beds were washed by shaking the flasks with 5–7 glass beads for 30 min at 200 rpm. Viable counts of *E. coli* were determined by dilution plating on LB soft agar (0.5% agar) plates.

### Spore germination assay (Ancestors and lab-evolved *M. xanthus* isolates)

Each strain used for the germination assay had a specific sporulation efficiency, as measured using the sporulation assay mentioned earlier (see S2 Table for sporulation efficiencies). Based on this, we predicted the number of spores expected from the initial inoculum size of each strain used in the germination assays. To obtain a spore suspension of 10⁴ spores/mL for the ancestral isolate, spores from one starvation plate (100 µL of 5 × 10⁹ cells/mL inoculum per plate) were harvested in 1 mL of ddH₂O and serially diluted to the desired initial spore density. For clone 1 from the 15% regimen, spores from 10 starvation plates (100 µL of 5 × 10⁹ cells/mL inoculum per plate) were harvested in 1 mL ddH₂O to achieve an initial spore density of 10⁴ spores/mL. For clone 1 from the 1% regimen, spores from one starvation plate were harvested in 1 mL ddH₂O and serially diluted to the desired starting density of 10⁴ spores/mL.

To confirm that the expected number of spores was the same as the actual number used in the germination assay, spore suspensions were plated after serial dilution on CTT soft agar (0.5% agar) plates, incubated at 32°C for 3–4 days, and colonies were counted. These counts were used to determine the initial spore numbers inoculated in the germination assays (T₀ spore counts).

For germination assays analyzing the lab-evolved isolates and their ancestors, *E. coli* was used as the only nutrient source. *E. coli* cultures were obtained by inoculating a single colony into LB medium, followed by overnight incubation at 200 rpm and 32°C. The *E. coli* cultures were then washed once with TPM buffer and resuspended to an O.D. _600 nm_ of 0.1 in TPM buffer. A 300 µL aliquot of *E. coli* culture (O.D. _600 nm_ 0.1) was inoculated with 10⁴ spores in 1.5 mL microcentrifuge tubes and incubated at 32°C for 4 hours. After incubation, 100 µL of the co-culture was transferred to 900 µL ddH₂O, heated at 50°C for 2 hours, and sonicated twice for 10 s (Amplitude: 25, Pulse: 10 s ON, 10 s OFF, 10 s ON). A 100 µL aliquot of the sonicated culture was dilution-plated onto CTT soft agar (0.5% agar) plates.

The heat treatment and sonication kill spores that have germinated into cells and have lost resistance to sonication and heat. Therefore, the colonies appearing on the CTT soft agar plates represent the number of spores that did not germinate within 4 hours (T₄). The difference between the initial spore count and the final spore count was used to calculate germination efficiency.


$$\text{Germination efficiency }=\;(\frac{Initial\;number\;of\;spores\;-\;Final\;number\;ofspores\;after\;four\;hours}{Initial\;number\;of\;spores})\times \text{1}\text{0}\text{0}$$

### Spore germination assay (Natural isolates of *M. xanthus*)

Since *M. xanthus* spore germination (for the isolates used in this study) occurs relatively quickly, the germination assays described above are time-sensitive and not ideal for high-throughput analysis of multiple strains. Therefore, to analyze the germination efficiencies of 13 natural isolates of *M. xanthus*, we used an Alamar dye-based assay (alamarBlue Cell Viability Reagent, cat. No DAL1025). This assay works on the principle of color change in the Alamar dye under a reducing environment, which indicates the germination of spores into metabolically active vegetative cells.

For the germination assays, 270 µL of spore suspensions (10⁶ spores/mL) were incubated with 30 µL of Alamar blue dye (10x stock concentration) in 96-well microtiter plates. The plates were incubated at 32°C in a plate reader for 4.5 hours, during which fluorescence intensities (excitation at 550 nm, emission at 590 nm) were measured at 5 min’ intervals for 4.5 hours.

To address the time constraints associated with the previous germination assays, the impact of spore density on the germination efficiency of ancestor spores was also evaluated using the Alamar dye-based assay. Spores from the ancestor clones were initially adjusted to a density of 5 × 10⁷ spores/mL and then serially diluted to achieve different starting densities, following the same Alamar dye-based assay protocol.

### Growth curve assay

*M. xanthus* isolates were revived from glycerol stocks and grown in 8 mL of CTT liquid medium in 50 mL conical flasks at 32°C with shaking at 200 rpm until they reached an O.D. _600 nm_ of 0.2–0.8. The cultures were then centrifuged at 5,000 rpm for 20 min at 25°C, and the cell density was adjusted to 5 × 10⁹ cells/mL using TPM buffer. A 10 µL aliquot of the density-adjusted culture was transferred to 1 mL of CTT liquid in a 48-well microtiter plate (Tarsons, Cat. No. 980051), sealed with a transparent cover (Bio-Rad, MSB1001), and incubated in a Tecan plate reader (Infinite M Nano) at 32°C for 48 hours. The O.D. _600 nm_ of the *M. xanthus* cultures was measured every 5 min, with shaking for 10 s between readings for aeration.

For the vegetative cell density assays, different starting densities of *M. xanthus* cultures were incubated in 48-well microtiter plates at 32°C for 72 hours, and the O.D. _600 nm_ was measured at regular intervals as described above to demonstrate the effect of cell density on growth.

### Casein hydrolysis assay

*M. xanthus* clonal isolates of relaxed and stringent bottleneck regimes along with the ancestor population were revived from freezer stocks by spotting on CTT agar plates (1.5%) and incubated at 32°C for 3–4 days. From these plates, a portion of *M. xanthus* swarm were inoculated in 8 mL of CTT liquid medium in 50 mL conical flasks at 32°C with shaking at 200 rpm until they reached an O.D. _600 nm_ of 0.3–0.6, followed by adjustment of cell density to 5 x 10^9^ cells/ml which was diluted to 1:9 ratio in TPM liquid medium to get a cell density of 5 x 10^8^ cells/ml. Then, 150 μL of this density adjusted primary cultures were used to initiate respective secondary cultures, and once they reached an O.D. _600 nm_ of 0.4–0.6, the culture supernatants were harvested and sterilized using 0.2 μm syringe filters. Serial dilutions of culture supernatants were prepared in TPM liquid medium. Then, 50 μL of 8-fold diluted culture supernatants were added to 100 μL succinylated casein substrate provided with the Thermo Fisher Scientific Pierce colorimetric protease assay kit (cat. No. 23263) along with appropriate buffer controls. Ninety-six well-plate wells containing either supernatant and casein or buffer and casein were incubated at room temperature for 20 min then added with 50 μL TNBSA reagent followed by incubation for another 20 min at room temperature. The protease present in the supernatant hydrolyzes casein such that the trinitrobenzene sulfonic acid, TNBSA reacts with free amino terminal groups forming TNB-peptide adducts, which is measured at absorbance value of 450 nm. A higher absorbance at 450 nm indicates higher protease concentration in the supernatant. The standard curve was derived using trypsin protease activity and the respective concentration of protease concentration for test samples was quantified.

### Development competition assay

For different types of development competition assays mentioned in the paper, the respective strains were revived from glycerol stocks (described above) and were then cultured independently in CTT liquid medium. When the *M. xanthus* cultures reached an O.D. _600 nm_ of 0.2–0.8, the cells were pelleted by centrifugation (5,000 rpm, 20 min, 25°C) and then the cell densities were adjusted to 5 × 10⁹ cells/mL in TPM buffer. A 60 µL aliquot of the density adjusted culture was mixed with the 60 µL aliquot of the density adjusted culture of the ancestor GJV2 strain, unless otherwise stated. A 100 µL aliquot of this 1:1 mixed culture was spotted onto the starvation medium, TPM hard agar (1.5% agar) plates, while A 100 µL aliquot of each density adjusted mono-strain cultures were spotted as the controls. The co-culture as well as the mono-culture plates were incubated at 32°C for 3 days. After this 72 hours incubation, the fruiting body spots were harvested using a sterile scalpel, resuspended in 1 mL ddH₂O, sonicated twice for 10 s (Amplitude: 25, Pulse: 10 s ON, 10 s OFF, 10 s ON), and dilution-plated on CTT soft agar (0.5% agar) with and without rifampicin (rifampicin antibiotic is used to get the CFU counts of only the rifampicin resistant strains, including ancestor GJV2).

We used the following calculation to find out the relative spore productivity while in competition, mentioned as Wij. Cij calculation is used as an indicator to understand the effect of the mixed clones on each other’s performance.

The log-transformed sporulation efficiency of strain i and strain j in respective pure cultures are;


Di=log10[Ni(t3)/Ni(t0)]

Dj=log10[Nj(t3)/Nj(t0)]

where N(t0) = population size of strain i/j as vegetative cells before starvation and N(t3) = the viable population size of spores of strain i/j after 3 days of starvation.

Sporulation efficiency of strain i in the presence of strain j while in competition is given as;


Dij=log10[Ni(j,t3)/Ni(j,t0)]

Sporulation efficiency of strain i in the presence of strain j while in competition is given as;


Dji=log10[Nj(i,t3)/Nj(i,t0)]

The relative difference in sporulation efficiency of two strains when they are co-cultured is given as;


Wij=Dij−Dji

The effect of mixing of strain i and j on the sporulation efficiency of i is given as;


Cij=Dij−Di

During the evolved clone versus GJV2 ancestor development competition, as mentioned above, we mixed the density adjusted cultures of evolved clone and GJV2 ancestor at 1:1. Their respective mono-culture spots were served as the controls. We calculated, the relative spore productivity (Wij) and Cij for each competitions performed.

During intrapopulation competitions between the good sporulator (C12) and bad sporulator (C3) of 15%, the respective density adjusted cultures were mixed at 1:1 and were spotted TPM hard agar to induce starvation. Once again, their respective mono-cultures were used as controls. Prior to this particular competition experiment, we had to raise rifampicin resistant version of either C3 or C12, such that we could differentiate rifampicin resistant clones from nonresistant counterpart. We incubated C3 clone CTT-liquid grown culture in rifampicin added CTT-soft agar media for a duration of two weeks and picked the appeared resistant colonies. We setup competition experiment between the raised rifampicin strains of C3 against the original nonresistant C3 to verify the neutrality of the derived strains.

For the allele exchange strains’ competition, these strains and ancestor GJV2 strain were cultured and were density adjusted to 5 × 10⁹ cells/mL in TPM buffer. The allele-exchanged strains were then mixed with GJV2 at 1:1 ratio and were spotted onto the TPM hard agar plates. These plates were incubated for 3 days along with their respective mono-culture controls.

Post 3 days of incubation at 32°C, all co-culture and mono-culture spots were harvested using a sterile scalpel to a 1 mL ddH₂O, sonicated twice for 10 s (Amplitude: 25, Pulse: 10 s ON, 10 s OFF, 10 s ON), and dilution-plated on CTT soft agar (0.5% agar) with and without rifampicin. The GJV2 and the C3 strain used in the competition harbor rifampicin resistance, and they appeared in both antibiotic and nonantibiotic plates. The nonantibiotic added CTT soft agar media had the nonresistant strain along with the rifampicin resistant strain. So, depending on the specific competition experiment mentioned above, colonies on plates with rifampicin reflect either the population size of GJV2 (ancestor) or C3 strain, while the plates without rifampicin represent the total number of spores (GJV2/ C3 + nonresistant isolate).

### Life cycle competition assay

GJV2 (a rifampicin-resistant version of GJV1), evolved, and ancestor clone cultures were revived from glycerol stocks as described above. *M. xanthus* cultures with an O.D. _600 nm_ of 0.2–0.8 were centrifuged (5,000 rpm, 20 min, 25°C), and the cell densities were adjusted to 5 × 10⁹ cells/mL using TPM buffer. A 100 µL aliquot of the density-adjusted evolved isolate was mixed with 100 µL of density-adjusted GJV2 culture, and 200 µL of the cocultures were spotted onto TPM hard agar (1.5% agar) plates and propagated for two life cycle competition cycles using the same protocol as the evolution experiment. However, the cultures were not subjected to any bottleneck events.

At the end of the third round of development, the fruiting body spots were harvested using a sterile scalpel, resuspended in 1 mL ddH₂O, sonicated twice for 10 s (Amplitude: 25, Pulse: 10 s ON, 10 s OFF, 10 s ON), and dilution-plated on CTT soft agar (0.5% agar) with and without rifampicin. GJV2 is a rifampicin-resistant variant of GJV1, so colonies on plates with rifampicin reflect the population size of GJV2 (ancestor), while the plates without rifampicin represent the total number of spores (GJV2 + evolved isolate).

### Whole genome sequencing of ancestors and evolved clones

The ancestors and evolved clones (from the T_10_ cycle) of the experimental evolution were directly inoculated from their respective glycerol stocks into 8 mL of CTT liquid in 50 mL conical flasks. The cultures were grown to an O.D. _600 nm_ of 0.4–0.8, centrifuged at 5,000 rpm for 10 min at 25°C, and the cell pellets were used for genomic DNA isolation. DNA was extracted using Qiagen’s Genomic DNA Extraction Buffer Kit (cat. no. 19060) and 20/G genomic tips. The eluted DNA was stored in 30 µL of autoclaved Milli-Q water.

The quantity of the extracted DNA was initially checked using a Nanodrop spectrophotometer and later at the sequencing facility (Macrogen, South Korea) using a Qubit fluorometer. Sequencing was performed on an Illumina HiSeq4000 system using the TruSeq Nano DNA Kit (350) in paired-end mode, generating 150 bp read lengths. The samples were prepared following the NGS library preparation workflow.

Read quality was assessed using FastQC, and Illumina-specific adapters or primers were trimmed from the reads using Trimmomatic v0.40 with the following parameters: ILLUMINACLIP: TruSeq3-PE.fa:2:30:10:2 LEADING:3 TRAILING:3 MINLEN:36. For mutation calling, the processed reads were mapped to a modified version of the *M. xanthus* DK1622 genome (available on NCBI with refseq: NC_008095 [46]) using the breseq pipeline from Barrick’s lab [83]. We used default clonal analysis parameters described in the breseq documentation, with bowtie2 [84] as the alignment tool against the reference genome.

### MXAN_1093 allele exchange for ancestor and 15% evolved clone with 15% mutant allele and wild-type allele respectively

1.1 kbp fragment for MXAN_1093 was amplified using the following primer pair

Primer 1 (forward): 5′ ACATCACCGAATCCGAGAGC 3′

Primer 2 (reverse): 5′ GACTGATAGGCGCGGTACTC 3′

such that the mutation of interest comes at the middle region of the final amplified fragment.

We amplified both MXAN_1093 wild-type allele and MXAN_1093 15% mutant allele using the above primers and completed the following two-step cloning procedure for both alleles of interest.

a) **Cloning 1: insert cloning to pCR-Blunt**

The PCR amplified fragment was ligated into the cloning vector pCR-Blunt (Invitrogen), which has a kanamycin resistance gene. The ligation mixture was then transformed into DH5α competent *E. coli* strain and the transformants were selected on the LB-Kan plates. The plasmid DNA was isolated from the positive colonies and was subjected to restriction digestion with BamH1 and EcoRV and the success of cloning was confirmed by checking the fragment size. The 1.1 kbp fragment was gel extracted, column purified (Qiagen) and used for further cloning into pBJ113 plasmid which facilitates its exchange in *M. xanthus* with native allele in its genome. Additionally, these *E. coli* clones were frozen for long-term storage.

b) **Cloning 2: insert cloning to pBJ113**

The isolated 1.1 kbp insert fragment from the above step with flanking BamH1 and EcoRV sites was ligated into BamH1 and HincII digested pBJ113 plasmid [85]. (pBJ113 plasmid digestion with BamH1 and HincII generated two fragments. The larger fragment from this digestion was used for the above-mentioned cloning since this fragment contains a kanamycin resistance gene (selection for plasmid integration in *M. xanthus* target genome) and a galK gene (this gene product prevents growth on galactose and used as a screening tool to select *M. xanthus* colonies which can grow on galactose plate post successful plasmid excision).

The pBJ113 plasmid containing the insert was then transformed into DH5α competent *E. coli* strain and the transformants were picked from LB-Kan plates. The correct insert size was checked using EcoRI digestion and the positive clones were frozen for long-term storage.

c) **Electroporation and two step selection for true allele-exchanged transformants**

The ancestor *M. xanthus* strain and evolved 15% *M. xanthus* strain (both are kanamycin sensitive strains) were electroporated (conditions) respectively with pBJ113 derived plasmid-with-1093-mutant allele insert and pBJ113 derived plasmid-with-1093-WT allele insert. The kanamycin resistant transformants were selected on CTT kanamycin soft agar plates.

The kanamycin resistant transformants were later grown in nonselective CTT-liquid medium to 0.4–0.5 O.D._600_ nm, diluted and plated on 1% galactose CTT soft agar for secondary selection. Only the cells with genomes from which the plasmid spontaneously excised (absence of galK gene) could grow on galactose CTT soft agar, and we collected as many colonies as possible and inoculated them into CTT-liquid medium and were stocked for long-term storage. The stocked cultures were then streaked on 1% galactose CTT hard agar to get individual colonies. Colonies were screened for their change of phenotype by culturing and spotting on TPM hard agar plates after adjusting the density to 5x10^9^ cells/mL. The spots were checked post 3 days of incubation at 32°C and were screened for positive phenotype which is good fruiting body structure in 15% clone background (suggesting the native mutant allele got replaced by wild-type 1093 allele) and bad fruiting body structure in ancestor background (suggesting the native wild allele got replaced by mutant 1093 allele of 15%)

Three clones from each successful allele exchange categories were stored, and the PCR amplified fragments post colony PCR were sent for sequencing. To confirm the gain and loss of fruiting body phenotype quantitively in respective conditions and to see the effect of exchanged alleles on the strain’s competitive fitness, spore productivity assays were performed (see Materials and methods section for spore productivity and developmental competition) with the selected three clones each.

### Population-wide computational model mimicking the synthetic life cycle experimental evolution of *M. xanthus* conducted in the lab

Table 1 provides an overview of the parameters used in the model.

**Table 1:** Overview of the parameters used in the model.

Parameter	Description	Range
β	Bottleneck size	0.01 or 0.15
γ	Cost of cooperation during C–C interactions during sporulation	0–1
ε	Degree of privatization of goods during germination	0–1

The simulation started with 1,000,000 cells. The number of cycles in the simulation is 50. The cycle count is maintained at 50 to ensure the stabilization of spore productivity and germination efficiency patterns over these iterations. The sporulation productivity and germination efficiency are computed at the end of the 10th (number of cycles done in the lab evolution) and 50th cycle. At the start of the 1st cycle, each cell is designated with its sporulation and germination ability based on the truncated normal probability distribution (although the values will lie between 0 and 1). The first cycle is the same for both the system—1% population bottleneck and 15% population bottleneck, as the bottlenecks are introduced after germination (in line with the experiments). For the first cycle, the process of sporulation is simulated, where cells are selected based on the individual ability to sporulate (see S3 Table). The parameter P(i) captures the probability of sporulation of bacterial cells in the presence of other bacterial cells. Similarly, S(i) indicates the individual ability of sporulation of bacterial cells. From the 2nd cycle onwards (i.e., after introducing population bottlenecks), the cells are selected for sporulation based on the interaction between the exploiters and cooperators. The cells having sporulating ability more than or equal to 0.5 are classified as “cooperators,” and the rest are classified as “exploiters”. Therefore, the proportion of cooperators (p(c)) and proportion of exploiters (p(e)) are computed. Next, the probability of cooperator-cooperator (C–C) interactions, cooperator-exploiters (C–E) interactions, and exploiter-exploiter (E–E) interactions are computed (see S3 Table). After that, randomly, cells are selected from the cooperators and exploiters array, which will undergo these interactions. In the model, the ‘gamma (γ)’ factor encapsulates the cost of cooperation during sporulation. Its values range from 0 to 1. The values closer to 0 will indicate 100% cost associated with the cooperation; thus, cooperation yields zero benefits. On the other hand, values closer to 1 indicate a negligible cost of cooperation. Also, it means cells don’t undergo any interaction with each other; thus, there is no cost of cooperation. Cells undergoing C–C interaction bear a cost (e.g., production of excess public goods). Therefore, their cooperation do not show an additive effect. The total number of spores formed is computed by summating all the cooperator cells that undergo C–C interactions, then multiplied by a factor ‘γ’ (see pay-off matrix in S4 Table). Bacterial cells undergoing the exploiter-exploiter interaction under a 15% bottleneck system yield no spore formation. Therefore, the payoff of E–E interactions (N) is zero.

For capturing the dynamics of exploiter cells around cooperators, i.e., C–E interactions, the cells are selected randomly from the rest of the array. It has been taken care of that a particular cell is selected for only one type of interaction. Now, in order to compute the payoffs for cooperators and exploiters cells from C–E dynamics, first their average ability of sporulation is computed. The means indicates the average capability of cells to produce the public goods necessary for sporulation. Thereafter, the difference(D) between these means is computed (see pay-off matrix, S4 Table). Thereafter, the sharing of public goods takes place between the exploiters and cooperators based on the rule given in S4 Table. The factor ‘*n*’ decides the degree of sharing, i.e., if *n* = 1, then all the public goods produced by cooperators will be taken away by exploiters. And if *n* = 1, it will indicate that extra goods are produced and shared, which does not make biological sense. Therefore, the value of ‘*n*’ is greater than or equal to 1. In the simulation, the value of ‘*n*’ used is 50. In this case, a specific amount of the public goods is shared between cooperators and exploiters. The extra goods the exploiters obtain are equally divided among all the exploiter cells. Similarly, equal goods are removed from the cooperator cells (i.e., changes in individual cells’ ability to sporulate). All the cells with a sporulation ability of more than 0.5 are selected afterward. During this selection, all the exploiters are selected whose ability to sporulate after interaction becomes more than 0.5 (i.e., enough public goods are available to sporulate), and all the cooperators are selected whose ability is still above 0.5.

In the model the payoffs are as follows.


$$\begin{array}{c} K\left(c\right)\;=\gamma \;.\;\frac{\text{1}}{\text{2}}\;\sum {\mathrm{\backslash nolimits}}_{i=\text{1}}^{m}S\;(i),\;L\left(c\right)=\sum {\mathrm{\backslash nolimits}}_{i=\text{1}}^{p}Sc(i{)}^{\prime },\;M(e)=\sum {\mathrm{\backslash nolimits}}_{i=\text{1}}^{q}Se(i{)}^{\prime }\text{ and }N\;(e)\;=\;\text{0}\; \end{array}$$

The results show that the payoffs are in the order; K > M > L > N.

The germination process is simulated where the average germination ability of the cells is computed, and based on it, the cells are selected for the next phase. In the first cycle of the process, the bacterial cells undergoing germination are selected based on the average ability of all the cells to undergo germination. The degree of public good privatization in the germination phase is regulated by the factor – ‘epsilon (ε).’ The value of the ε factor ranges between 0 and 1. In the first cycle, it is kept at 0.8 (low degree of privatization). After germination, the cells are subjected to a bottleneck event in the model where randomly, 15% or 1% cells are randomly selected for the next phase of the experimental cycle. Following cell selection, the chosen cells enter a growth phase characterized by division, with each cell undergoing binary fission until the population reaches 1 million. During this growth phase, cells undergo mutations impacting their germination and sporulation abilities. Mutations occur randomly, with germination ability potentially increasing or decreasing within the range of [−0.1, 0.1], drawn from a uniform distribution. To introduce trade-offs between germination and sporulation, the sporulation ability negatively correlates with germination (correlation coefficient = −1.15). Thus, if germination ability increases due to mutation, sporulation ability decreases, and vice versa. Importantly, measures are in place to ensure that both germination and sporulation abilities remain within the range of [0, 1]. Cells that breach this boundary condition in either ability are considered to have undergone a lethal mutation and are removed from the cell population. This stringent control mechanism maintains the integrity of the simulated microbial population and accurately represents evolutionary dynamics in response to environmental pressures. At the conclusion of the simulations, spore productivity and germination efficiency are assessed as critical metrics of evolutionary success. Spore productivity, indicative of the population’s reproductive output, is calculated as the normalized logarithm of the ratio between the sporulation rates of the evolved and ancestral populations. Similarly, germination efficiency, a measure of the population’s ability to initiate growth from spores, is determined as the normalized logarithm of the ratio between the germination rates of the evolved and ancestral populations.

### Statistical analysis

R (version 4.3.1) was used to conduct all the data analyses and for plotting. Normality of the data was checked using Shapiro–Wilk’s test, and Levene’s test was used to check the homogeneity of variance between the samples. Depending on these test results parametric or nonparametric was chosen appropriately. The figure legends include a description of the statistical test. Every experiment was run in three or more independent replicates separated in different time blocks. The computational model was created employing MATLAB language using the MATLAB 2020b version. Matlab R2024a was used to make the 3-D graph.

## Supporting information

S1 Fig Fruiting body spots of the stringent (1%) and relaxed (15%) populations and clones are morphologically different from each other and the ancestor. Representative images of the TPM hard agar (1.5% agar) plates post 3 days of incubation of 100 µL of *M. xanthus* 5 x 10^9^ cells/mL density cultures are shown. Small white dots represent individual fruiting bodies after 3 days of incubation. (a) Populations from a relaxed regimen (15%) had fewer fruiting bodies, whereas populations from a stringent bottleneck regimen (1%) exhibited proficient fruiting body formation (small white dots). (b) Similar to population level observation, the clones isolated from the D15 line (representative line from relaxed regime) were less efficient at fruiting body formation, whereas the clones from D1 (representative line from stringent regime) exhibited proficient fruiting body development.
(TIF)

S2 Fig Consistent with their slower growth in CTT liquid media, 15% evolved clones showed significantly low protease secretion compared to both ancestor and 1% evolved clones. Data shown is the casein hydrolysis assay performed on culture supernatants of 15% and 1% evolved clones grown in CTT liquid media. The absorbance value at 450 nm refers to the concentration of free amine groups released upon casein hydrolysis, to which TNBSA reagent reacted, following the addition of protease containing supernatant. A higher absorbance at 450 nm indicates higher protease concentration in the supernatant. The small black dots indicate each individual evolved clones from each evolved populations, while the big black dot refers to the mean across individual clones across three independent replicates and the error bar is for 95% confidence interval. (two-sample *t* test between means of 1% and 15% evolved clones, *t* = −3.0886, df = 3.186, *p*-value = 0.0496). The red dotted line indicates the mean absorbance for ancestor, with the ribbon with blue shade indicating a confidence interval of 95%. The data used to produce all figures are provided in S1 Data folder.
(TIF)

S3 Fig Social traits in *M. xanthus* are positive density-dependent. Dots represent per capita efficiency values of respective traits measured for three different colonies each of ancestors, 15% evolved and 1% evolved populations. Dashed lines are fitted linear regression and shaded area are 95% confidence interval (a) Sporulation efficiency for the ancestral strain GV1, 15% evolved and 1% evolved clones on starvation media (TPM-hard agar) is shown. The slope is significantly positive for ancestors and isolates derived from a 1% selection regimen. (*n* = 4) (ancestor: *R sq. *= 0.2304, *p*-value = 0.0023; 15%: *R sq. *= −0.002, *p*-value = 0.3927; 1%: *R sq. *= 0.5442, *p*-value* *= 1.318 × 10^−6^) (b) Vegetative growth rate for the ancestral strain GV1, 15% evolved and 1% evolved clones in CTT liquid is shown. The slope is significantly positive for ancestors and isolates derived from 1% and 15% selection regimen (*n* = 4). The growth rate is density-dependent in the ancestor, 15% and 1% regimen. (ancestor: *R sq. *= 0.5915, *p*-value < 1.457 × 10^−7^; 15%: *R sq. *= 0.2178, *p*-value = 0.0095; 1%: *R sq. *= 0.2521, *p*-value* *= 0.0036) (c) Per capita predation efficiency of ancestral *M. xanthus* clones and evolved clones from 15% and 1% treatments was measured as the growth of *E. coli* when it was co-cultured with *M. xanthus* at four different densities. The negative slope of the regression line indicates increasing predation efficiency with increasing density (*n* = 4). A significant negative slope in 15% shows that predation by these isolates is a density-dependent social trait. (*n* = 4) (ancestor: *R sq. *= 0.9525, *p*-value <* *2.2 × 10^−16^; 15%: *R sq. *= 0.9501, *p*-value <* *2.2 × 10^−16^; 1%: *R sq. *= 0.8766, *p*-value* *<* *2.2 × 10^−16^) (d) Germination efficiency of ancestral *M. xanthus* isolates was measured as a function of increasing spore density. Exit from dormancy and metabolic activity of the spores is measured as emission at 590 nm when spores are inoculated in nutrient-rich suspension with Alamar blue (*n* = 4) (ancestor: *R sq. *= 0.8857, *p*-value <* *2.2 × 10^−16^). For all traits analyzed ancestor clones exhibit positive cell density dependence. The data used to produce all figures are provided in S1 Data folder.
(TIF)

S4 Fig All three clones from the evolved 15% regimen have a point mutation at the 128th position of MXAN_1093 protein. Amino acid sequence alignment of ancestor to evolved 15% clone(s) for the DNA binding response regulator protein MXAN_1093 is shown above. The sequence alignment above indicates that at the 128th position of the protein the mutation resulted in the change of an aspartic acid to asparagine. The ancestor allele of MXAN_1093 is regulated by post-translation modification on aspartate residue at the 128th position in its response regulatory domain.
(TIF)

S5 Fig All three clones from the evolved 1% regimen had a frameshift mutation, which resulted in frameshift from 444th position onwards in MXAN_4899 protein. Amino acid sequence alignment of ancestor to evolved 1% clone(s) for the sigma 54-interacting transcriptional regulator protein MXAN_4899 is shown above. The highlighted protein segment indicates the frameshift occurred in the evolved 1% clone(s) in MXAN_4899 protein. Additionally, this shift in the protein-coding frame could result in the accessibility of a new stop-codon, which is likely to result in 485 amino acids long protein in 1% compared to the original length of 459 amino acids in the ancestor. The functional role of the helix shown in the box is unknown.
(TIF)

S6 Fig Computational model aligns with the empirical findings at specific combinations of parameter values demonstrating the role of cost of cooperation during sporulation (γ) and degree of privatization during germination (ε). Represents the 2-D deconstruction of the 3-D figure (Fig 5b) given in the main text. The Y-axis represents the germination/sporulation efficiency relative to the ancestor for a specific combination of γ and ε value. ε value is kept constant within each grid, varying γ from 0.6–0.98 in the x-axis. ε range varies from 0.4–0.9 from top-left to bottom-right grid. The data used to produce all figures are provided in S1 Data folder.
(TIF)

S7 Fig Sporulation and germination profile of cells at different phases of the life cycle along with the distribution of cooperators and exploiters cells during the sporulation phase of the cycle. (a and b) Graphs demonstrate the distribution of sporulation and germination abilities of ancestor cells, which is identical for both 1% and 15% systems. (c and d) Sporulation profile for the cells at the end of the germination process of the 10th cycle and Germination profile for the cells at the end of the germination process of the 10th cycle for 1% system (e and f) Sporulation profile for the cells at the end of the germination process of the 10th cycle and Germination profile for the cells at the end of the germination process of the 10th cycle for 15% system (g and h) Graphs shows the evolving proportion of cooperators and exploiter cells during the sporulation phase of the cycle for both 1% and 15% system from cycle 2–50. The data used to produce all figures are provided in S1 Data folder.
(TIF)

S1 Table Clones from the relaxed bottleneck regime show drastically reduced sporulation efficiency compared to the clones from both stringent and ancestor regimes. The table represents the sporulation efficiencies of ancestor, 12 clones of stringent (1%) and 11 clones of relaxed (15%) regimes. The sporulation efficiencies are calculated as the percentage of spores formed when 100 µL of 5 x 10^9^ cells/mL was allowed to sporulate on a TPM hard agar (1.5% agar) plate.
(DOCX)

S2 Table Distinct mutations in stringent and relaxed bottleneck evolution regimes. Whole genome sequencing was performed on representative clones from the stringent (1%) and relaxed (15%) regimens. All sequenced clones from stringent regimens had a frameshift mutation in one of the sigma-54-interacting transcriptional regulators (MXAN_4899). All sequenced clones from the relaxed regimen had a missense mutation in one of the DNA binding response regulator genes (MXAN_1093) [46,66]. Whole genome sequencing data is provided in figshare database DOI: https://doi.org/10.6084/m9.figshare.c.7975292.
(PPTX)

S3 Table Model description for the *M. xanthus* synthetic life cycle simulations. The table and the flowchart describe the successive events included in the simulations of a simplified version of life cycle lab evolution, which include the alternating 10 cycles of sporulation, germination and growth phases. Mutations were allowed during the growth phase. Simulations were repeated for different combinations of γ [0,1] and epsilon [0,1] values.
(DOCX)

S4 TableThe pay-off matrix for interactions during the sporulation phase.During the sporulation phase, the cooperators (sporulation efficiency ≥0.5) and exploiters (sporulation efficiency <0.5) were allowed to interact and share public goods. The pay matrix is calculated as above for each type of allowed interaction.(DOCX)

S1 Text Supplementary information. Description of social behaviors in *M. xanthus*.
(PDF)

S1 Data This zipped folder contains excel data files to reproduce figures Fig 2a-e, Fig 3a-d, Fig 4a-c, Fig 5a-d, S2 Fig, S3a-d Fig, S6 Fig, S7a-h Fig. This folder also contains R codes to reproduce the figures, Matlab code for running the life cycle simulation and Matlab code to reproduce Fig 5b.
(ZIP)

## Abbreviations

Cij: one-way mixing effect
GV1: focal ancestor
GV2: focal ancestor with a rifampicin resistance marker
IISc: Indian Institute of Science
IQR: interquartile range
SD: standard deviation
