# Supplementary figures and images for: Bottleneck size drives the evolution of cooperative traits in an aggregative multicellular myxobacterium
Source: PLoS Biol. 2026 Jan 6;24(1):e3003499. doi: 10.1371/journal.pbio.3003499 (PMC12773805; doi:10.1371/journal.pbio.3003499)

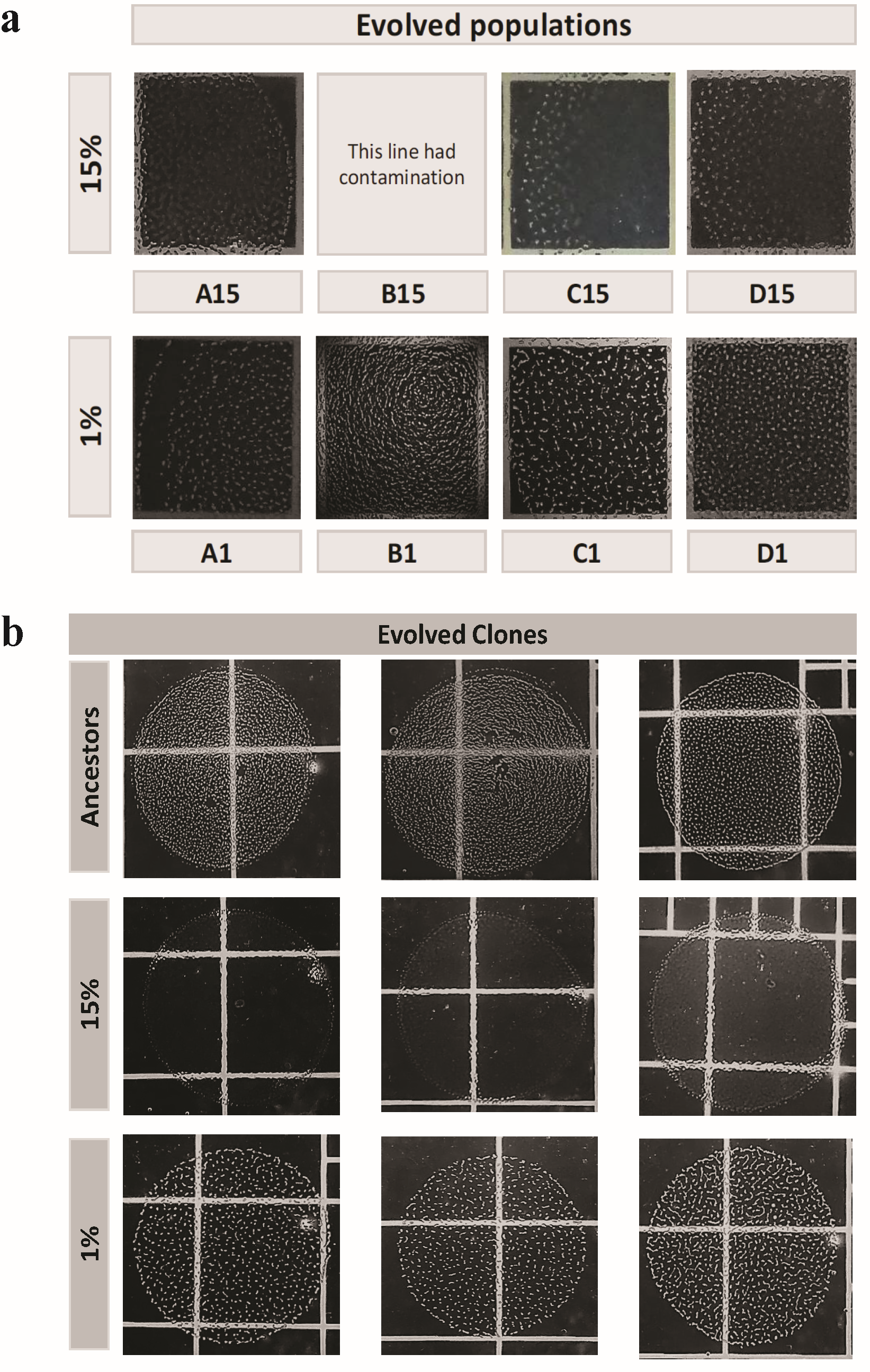

Supplement: S1 Fig — Representative images of the TPM hard agar (1.5% agar) plates post 3 days of incubation of 100 µL of M. xanthus 5 x 10^9^ cells/mL density cultures are shown. Small white dots represent individual fruiting bodies after 3 days of incubation. (a) Populations from a relaxed regimen (15%) had fewer fruiting bodies, whereas populations from a stringent bottleneck regimen (1%) exhibited proficient fruiting body formation (small white dots). (b) Similar to population level observation, the clones isolated from the D15 line (representative line from relaxed regime) were less efficient at fruiting body formation, whereas the clones from D1 (representative line from stringent regime) exhibited proficient fruiting body development. (TIF) [file pbio.3003499.s001.tif]

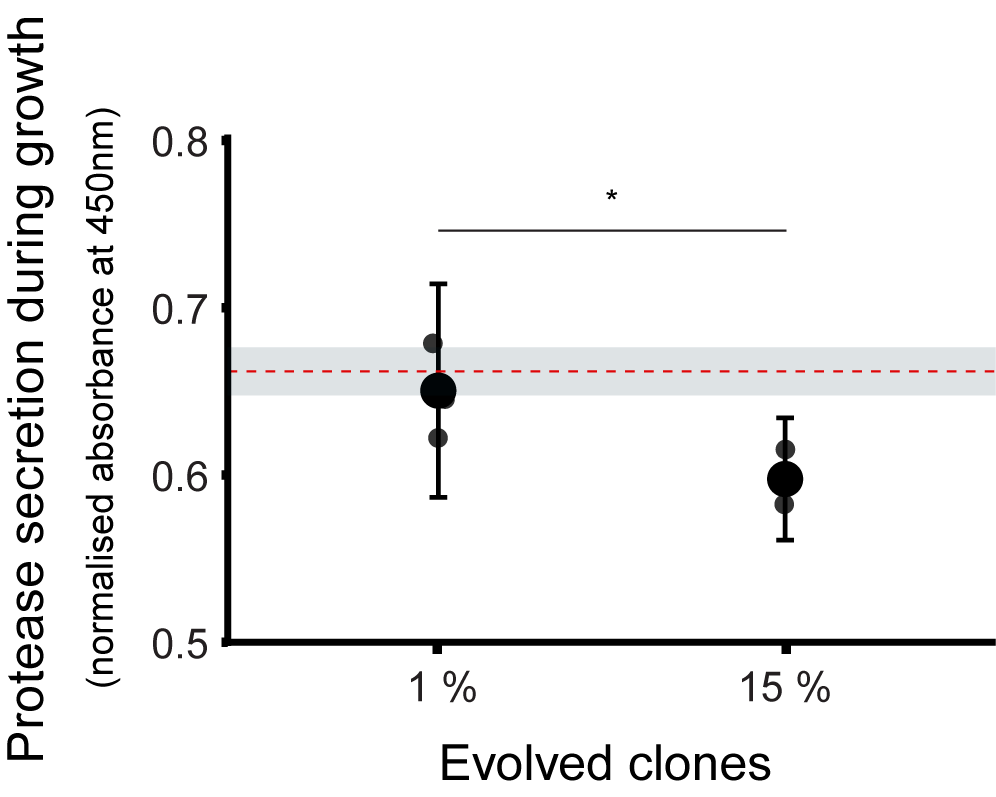

Supplement: S2 Fig — Data shown is the casein hydrolysis assay performed on culture supernatants of 15% and 1% evolved clones grown in CTT liquid media. The absorbance value at 450 nm refers to the concentration of free amine groups released upon casein hydrolysis, to which TNBSA reagent reacted, following the addition of protease containing supernatant. A higher absorbance at 450 nm indicates higher protease concentration in the supernatant. The small black dots indicate each individual evolved clones from each evolved populations, while the big black dot refers to the mean across individual clones across three independent replicates and the error bar is for 95% confidence interval. (two-sample t test between means of 1% and 15% evolved clones, t = −3.0886, df = 3.186, p-value = 0.0496). The red dotted line indicates the mean absorbance for ancestor, with the ribbon with blue shade indicating a confidence interval of 95%. The data used to produce all figures are provided in S1 Data folder. (TIF) [file pbio.3003499.s002.tif]

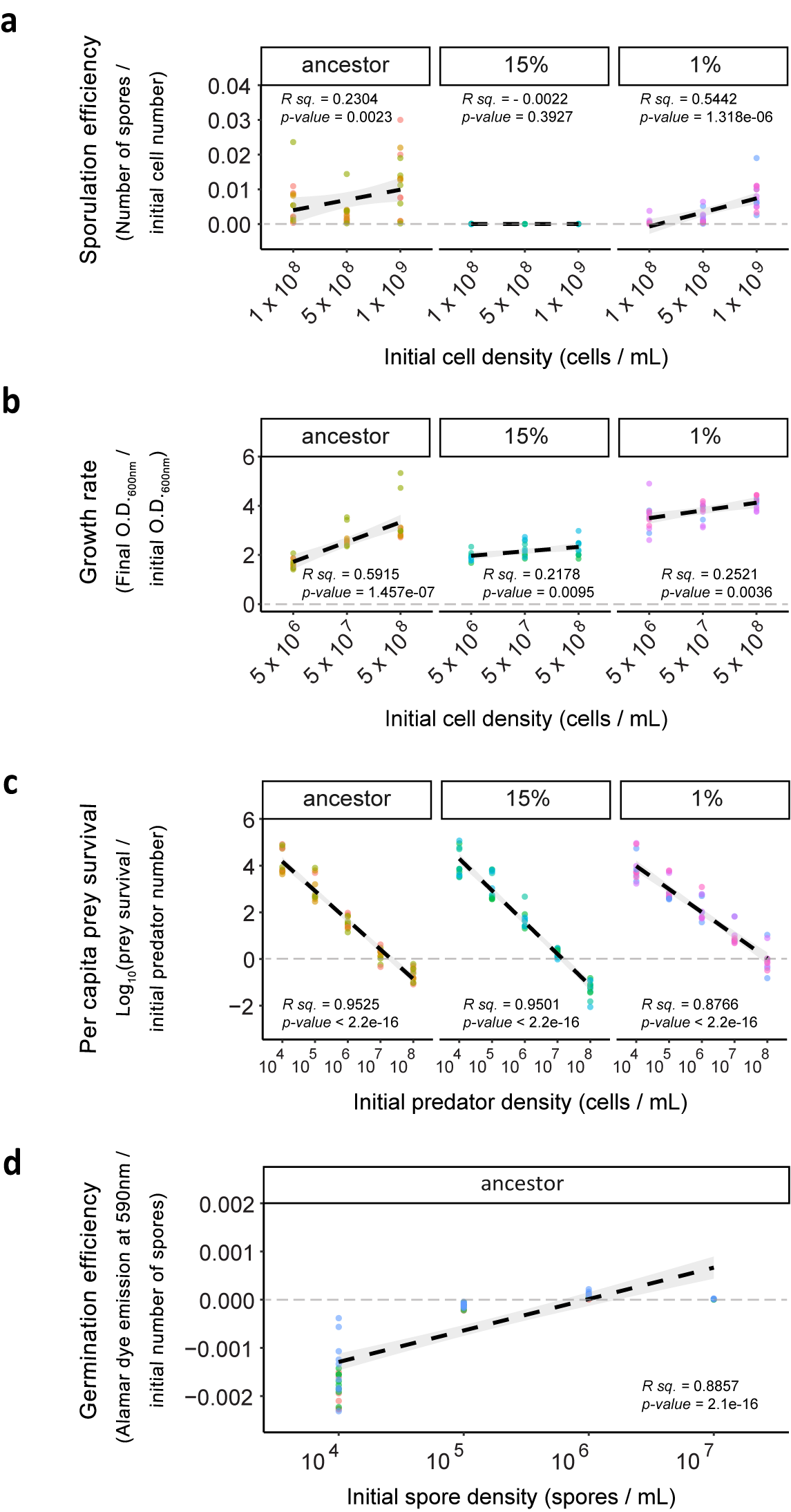

Supplement: S3 Fig — Dots represent per capita efficiency values of respective traits measured for three different colonies each of ancestors, 15% evolved and 1% evolved populations. Dashed lines are fitted linear regression and shaded area are 95% confidence interval (a) Sporulation efficiency for the ancestral strain GV1, 15% evolved and 1% evolved clones on starvation media (TPM-hard agar) is shown. The slope is significantly positive for ancestors and isolates derived from a 1% selection regimen. (n = 4) (ancestor: R sq. = 0.2304, p-value = 0.0023; 15%: R sq. = −0.002, p-value = 0.3927; 1%: R sq. = 0.5442, p-value = 1.318 × 10^−6^) (b) Vegetative growth rate for the ancestral strain GV1, 15% evolved and 1% evolved clones in CTT liquid is shown. The slope is significantly positive for ancestors and isolates derived from 1% and 15% selection regimen (n = 4). The growth rate is density-dependent in the ancestor, 15% and 1% regimen. (ancestor: R sq. = 0.5915, p-value < 1.457 × 10^−7^; 15%: R sq. = 0.2178, p-value = 0.0095; 1%: R sq. = 0.2521, p-value = 0.0036) (c) Per capita predation efficiency of ancestral M. xanthus clones and evolved clones from 15% and 1% treatments was measured as the growth of E. coli when it was co-cultured with M. xanthus at four different densities. The negative slope of the regression line indicates increasing predation efficiency with increasing density (n = 4). A significant negative slope in 15% shows that predation by these isolates is a density-dependent social trait. (n = 4) (ancestor: R sq. = 0.9525, p-value < 2.2 × 10^−16^; 15%: R sq. = 0.9501, p-value < 2.2 × 10^−16^; 1%: R sq. = 0.8766, p-value < 2.2 × 10^−16^) (d) Germination efficiency of ancestral M. xanthus isolates was measured as a function of increasing spore density. Exit from dormancy and metabolic activity of the spores is measured as emission at 590 nm when spores are inoculated in nutrient-rich suspension with Alamar blue (n = 4) (ancestor: R sq. = 0.8857, p-value < 2.2 × 10^−16^). For all traits an [file pbio.3003499.s003.tif]

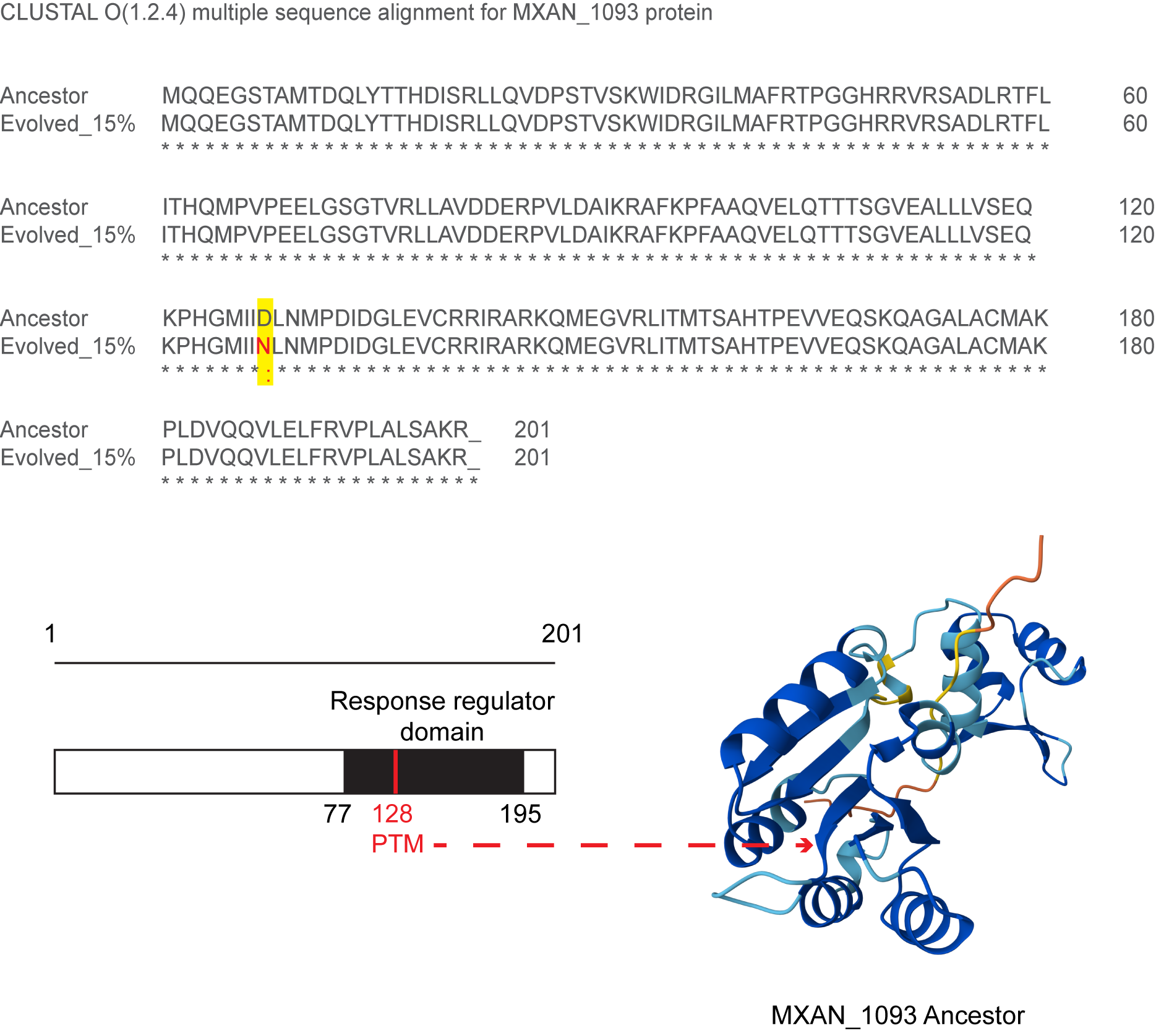

Supplement: S4 Fig — Amino acid sequence alignment of ancestor to evolved 15% clone(s) for the DNA binding response regulator protein MXAN_1093 is shown above. The sequence alignment above indicates that at the 128th position of the protein the mutation resulted in the change of an aspartic acid to asparagine. The ancestor allele of MXAN_1093 is regulated by post-translation modification on aspartate residue at the 128th position in its response regulatory domain. (TIF) [file pbio.3003499.s004.tif]

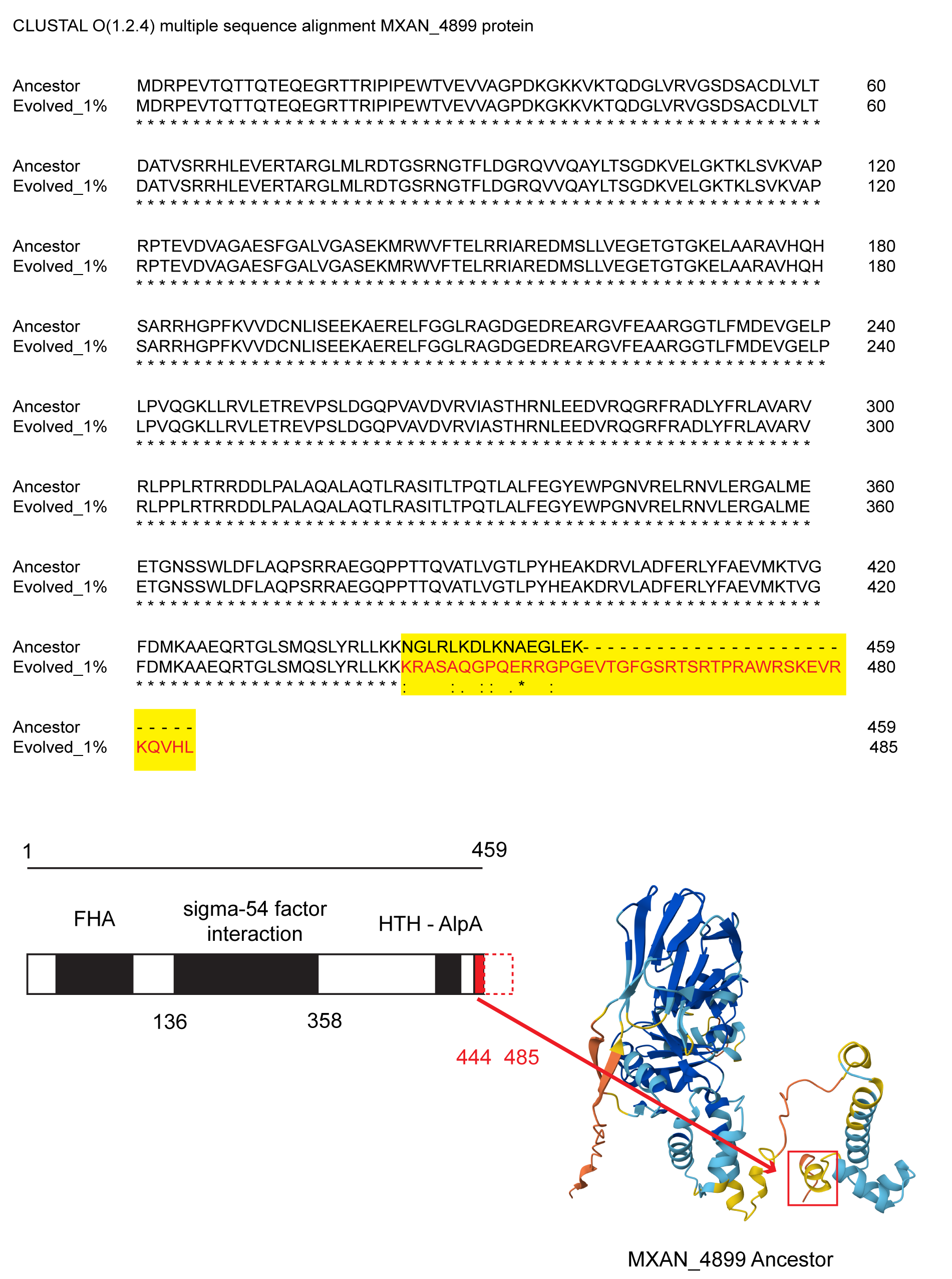

Supplement: S5 Fig — Amino acid sequence alignment of ancestor to evolved 1% clone(s) for the sigma 54-interacting transcriptional regulator protein MXAN_4899 is shown above. The highlighted protein segment indicates the frameshift occurred in the evolved 1% clone(s) in MXAN_4899 protein. Additionally, this shift in the protein-coding frame could result in the accessibility of a new stop-codon, which is likely to result in 485 amino acids long protein in 1% compared to the original length of 459 amino acids in the ancestor. The functional role of the helix shown in the box is unknown. (TIF) [file pbio.3003499.s005.tif]

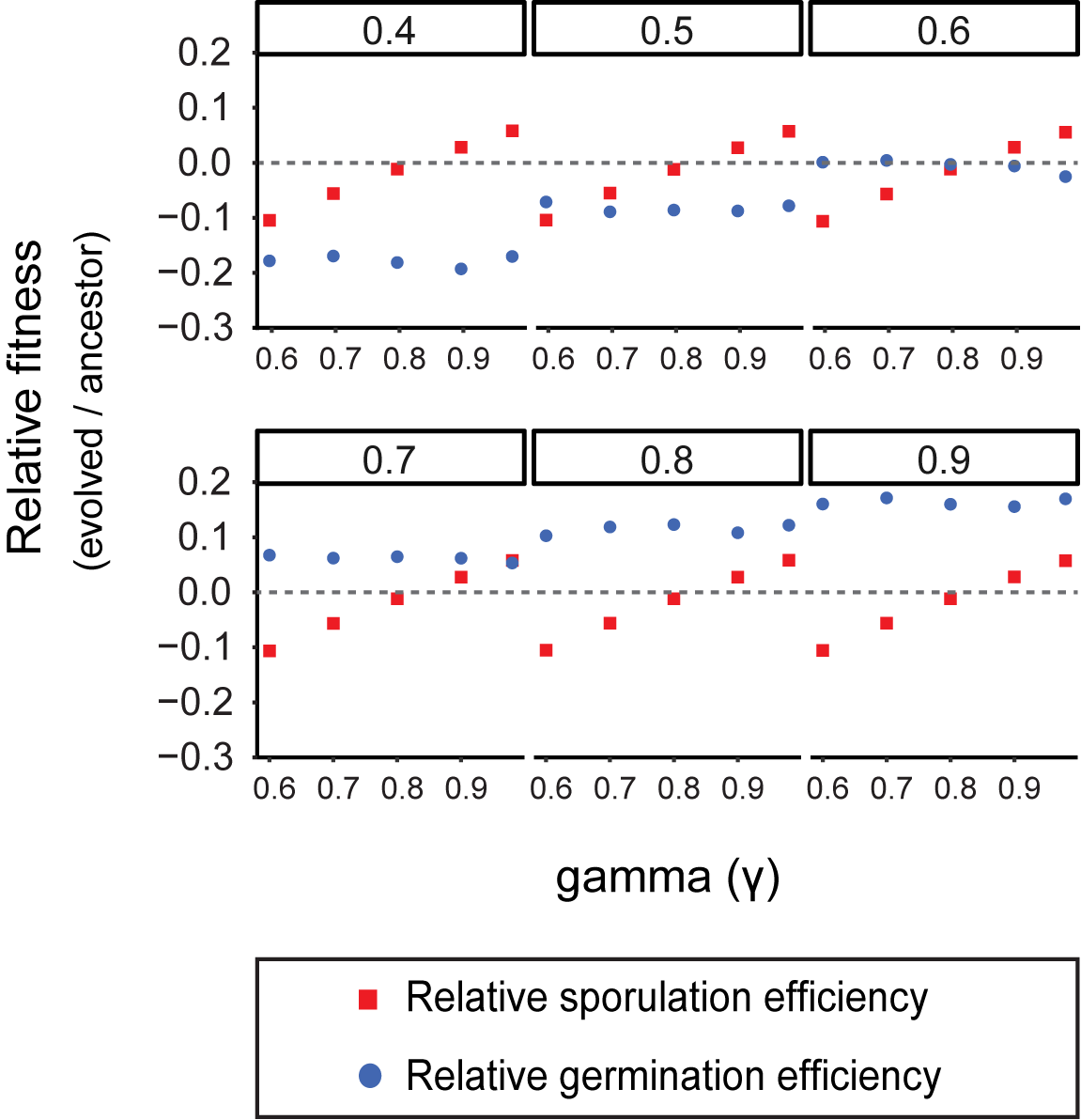

Supplement: S6 Fig — Represents the 2-D deconstruction of the 3-D figure (Fig 5b) given in the main text. The Y-axis represents the germination/sporulation efficiency relative to the ancestor for a specific combination of γ and ε value. ε value is kept constant within each grid, varying γ from 0.6–0.98 in the x-axis. ε range varies from 0.4–0.9 from top-left to bottom-right grid. The data used to produce all figures are provided in S1 Data folder. (TIF) [file pbio.3003499.s006.tif]

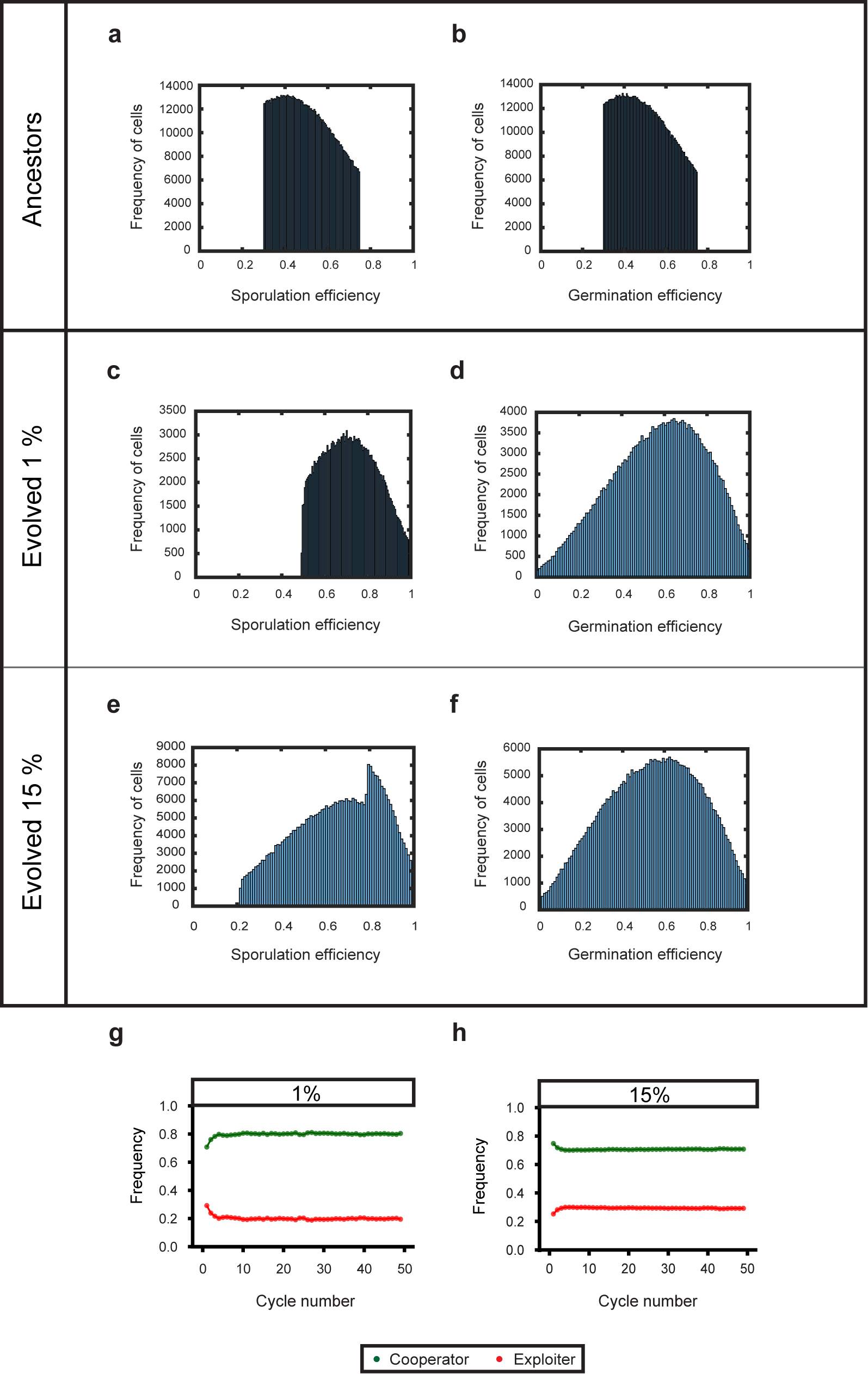

Supplement: S7 Fig — (a and b) Graphs demonstrate the distribution of sporulation and germination abilities of ancestor cells, which is identical for both 1% and 15% systems. (c and d) Sporulation profile for the cells at the end of the germination process of the 10th cycle and Germination profile for the cells at the end of the germination process of the 10th cycle for 1% system (e and f) Sporulation profile for the cells at the end of the germination process of the 10th cycle and Germination profile for the cells at the end of the germination process of the 10th cycle for 15% system (g and h) Graphs shows the evolving proportion of cooperators and exploiter cells during the sporulation phase of the cycle for both 1% and 15% system from cycle 2–50. The data used to produce all figures are provided in S1 Data folder. (TIF) [file pbio.3003499.s007.tif]
