# Supplementary material for: Bottleneck size drives the evolution of cooperative traits in an aggregative multicellular myxobacterium
Source: PLoS Biol. 2026 Jan 6;24(1):e3003499. doi: 10.1371/journal.pbio.3003499 (PMC12773805; doi:10.1371/journal.pbio.3003499)
Supplement: S1 Table — The table represents the sporulation efficiencies of ancestor, 12 clones of stringent (1%) and 11 clones of relaxed (15%) regimes. The sporulation efficiencies are calculated as the percentage of spores formed when 100 µL of 5 x 10^9^ cells/mL was allowed to sporulate on a TPM hard agar (1.5% agar) plate. (DOCX) [file pbio.3003499.s008.docx]

| **Regime** | **Clones** | **Sporulation efficiency (%)** | **Confidence interval (95 %)** |
| --- | --- | --- | --- |
|  |  |  |  |
| **Ancestor** | GV1 | 0.024667 | 0.014 |
|  |  |  |  |
| **1 %, D1 population** | C1 | 0.01311 | 0.01 |
|  | C2 | 0.00713 | 0.005 |
|  | C3 | 0.00664 | 0.003 |
|  | C4 | 0.00617 | 0.004 |
|  | C5 | 0.01585 | 0.004 |
|  | C6 | 0.0504 | 0.008 |
|  | C7 | 0.0598 | 0.016 |
|  | C8 | 0.0078 | 0.001 |
|  | C9 | 0.0474 | 0.007 |
|  | C10 | 0.05595 | 0.015 |
|  | C11 | 0.05785 | 0.017 |
|  | C12 | 0.02415 | 0.006 |
|  |  |  |  |
| **15 %, D15 population** | C1 | 0.00008 | 0 |
|  | C2 | 0.00047 | 0.0008 |
|  | **C3** | **0.00006** | **0** |
|  | C5 | 0.00171 | 0.0005 |
|  | C6 | 0.0007 | 0.0003 |
|  | C7 | 0.01295 | 0 |
|  | C8 | 0.0217 | 0.0159 |
|  | C9 | 0.00248 | 0.001 |
|  | C10 | 0.00351 | 0.0012 |
|  | C11 | 0.00492 | 0.0025 |
|  | **C12** | **0.06355** | **0.0189** |

S1 Table
