# Supplementary material for: Bottleneck size drives the evolution of cooperative traits in an aggregative multicellular myxobacterium
Source: PLoS Biol. 2026 Jan 6;24(1):e3003499. doi: 10.1371/journal.pbio.3003499 (PMC12773805; doi:10.1371/journal.pbio.3003499)
Supplement: S2 Table — Whole genome sequencing was performed on representative clones from the stringent (1%) and relaxed (15%) regimens. All sequenced clones from stringent regimens had a frameshift mutation in one of the sigma-54-interacting transcriptional regulators (MXAN_4899). All sequenced clones from the relaxed regimen had a missense mutation in one of the DNA binding response regulator genes (MXAN_1093) [46,66]. Whole genome sequencing data is provided in figshare database DOI: https://doi.org/10.6084/m9.figshare.c.7975292. (PPTX) [file pbio.3003499.s009.pptx]

## Slide 1
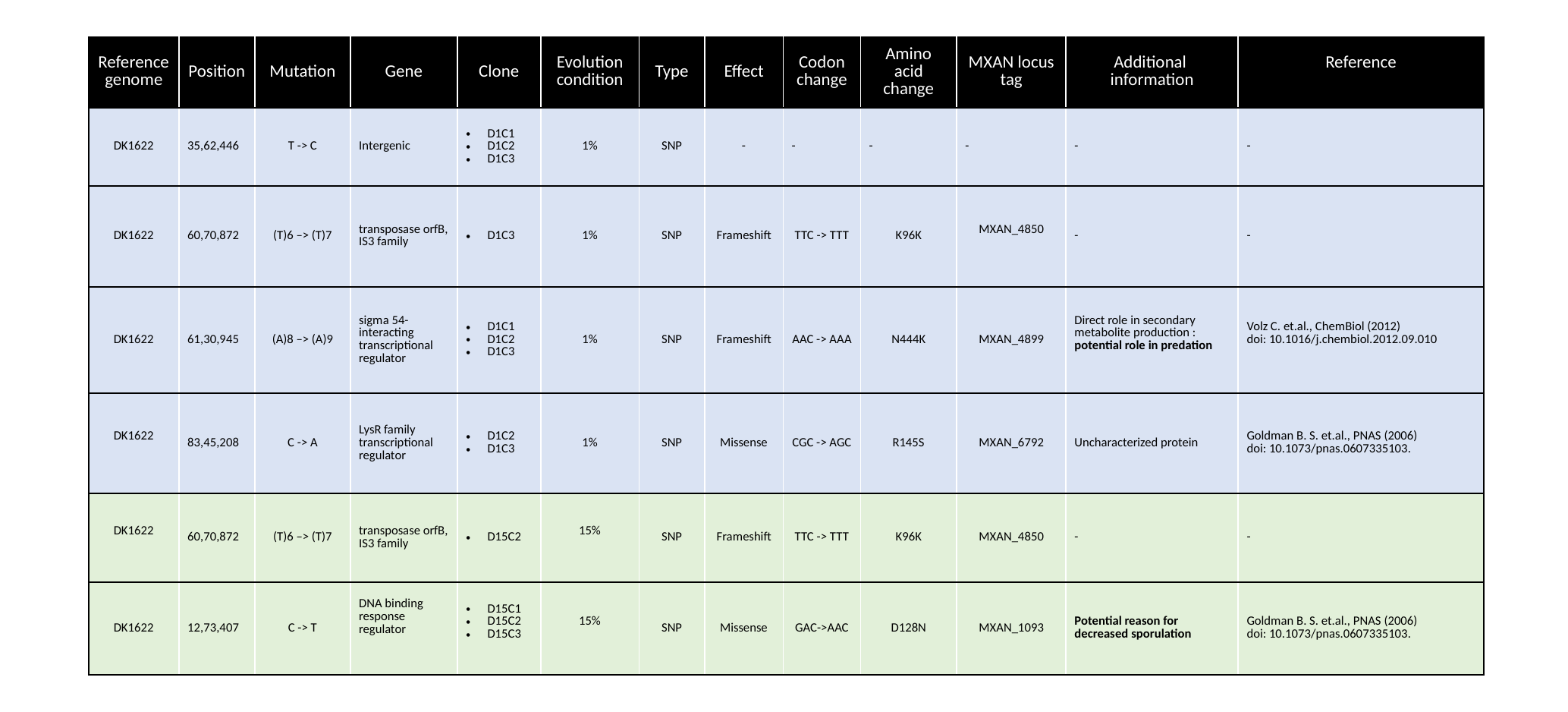

| Reference genome | Position | Mutation | Gene | Clone | Evolution condition | Type | Effect | Codon change | Amino acid change | MXAN locus tag | Additional information | Reference |
| --- | --- | --- | --- | --- | --- | --- | --- | --- | --- | --- | --- | --- |
| DK1622 | 35,62,446 | T -> C | Intergenic | D1C1 D1C2 D1C3 | 1% | SNP | - | - | - | - | - | - |
| DK1622 | 60,70,872 | (T)6 –> (T)7 | transposase orfB, IS3 family | D1C3 | 1% | SNP | Frameshift | TTC -> TTT | K96K | MXAN\_4850 | - | - |
| DK1622 | 61,30,945 | (A)8 –> (A)9 | sigma 54-interacting transcriptional regulator | D1C1 D1C2 D1C3 | 1% | SNP | Frameshift | AAC -> AAA | N444K | MXAN\_4899 | Direct role in secondary metabolite production : potential role in predation | Volz C. et.al., ChemBiol (2012) doi: 10.1016/j.chembiol.2012.09.010 |
| DK1622 | 83,45,208 | C -> A | LysR family transcriptional regulator | D1C2 D1C3 | 1% | SNP | Missense | CGC -> AGC | R145S | MXAN\_6792 | Uncharacterized protein | Goldman B. S. et.al., PNAS (2006) doi: 10.1073/pnas.0607335103. |
| DK1622 | 60,70,872 | (T)6 –> (T)7 | transposase orfB, IS3 family | D15C2 | 15% | SNP | Frameshift | TTC -> TTT | K96K | MXAN\_4850 | - | - |
| DK1622 | 12,73,407 | C -> T | DNA binding response regulator | D15C1 D15C2 D15C3 | 15% | SNP | Missense | GAC->AAC | D128N | MXAN\_1093 | Potential reason for decreased sporulation | Goldman B. S. et.al., PNAS (2006) doi: 10.1073/pnas.0607335103. |
