# Supplementary material for: Bottleneck size drives the evolution of cooperative traits in an aggregative multicellular myxobacterium
Source: PLoS Biol. 2026 Jan 6;24(1):e3003499. doi: 10.1371/journal.pbio.3003499 (PMC12773805; doi:10.1371/journal.pbio.3003499)

S3 Table

| **Cycle** | **Events** | **1 % transfer** | **15 % transfer** |
| --- | --- | --- | --- |
| Cycle 1 | Starting population size | 1,000,000 cells | |
|  | Sporulation efficiency | $\boldsymbol{P}\left( \boldsymbol{i} \right)\mathbf{=}\boldsymbol{S}\left( \boldsymbol{i} \right)$  Stored $\to S\left( i \right)temp$ | |
|  | Germination efficiency  $\varepsilon$ = Degree of privatization of goods during germination; [0-1]  0 – High privatization, 1- low privatization | $\mathbf{<}\boldsymbol{G}\mathbf{>}$ **=** $\boldsymbol{\varepsilon}\mathbf{.}$ $\frac{\boldsymbol{1}}{\boldsymbol{n}}\sum_{\boldsymbol{i}\mathbf{=}\boldsymbol{1}}^{\boldsymbol{q}} \boldsymbol{G}\mathbf{(}\boldsymbol{i}\mathbf{)}$**, ε = 0.8**  $\boldsymbol{P}\mathbf{(}\boldsymbol{i}\mathbf{) = <}\boldsymbol{G}\mathbf{>}$  Stored $\to G\left( i \right)temp$ | |
|  | Bottleneck event  β = Bottleneck size ; 1 % or15 % | β = 0.01 | β = 0.15 |
|  | Growth with mutations | Mutation rate = [-0.1,0.1]  Trade-off between germination and sporulation = $\boldsymbol{-1.15\pm0.02903}$  $S\left( i \right)temp\to S\left( i \right)temp$* ; $G\left( i \right)temp\to G\left( i \right)temp$* | |
| Cycle 2  onwards | Sporulation efficiency  p(e) = proportion of exploiters (E)  p(c) = proportion of cooperators (C)  $\boldsymbol{\gamma}$ = Cost of cooperation during C - C interactions during sporulation; [0,1]  **n** = Degree of sharing during sporulation; [0,1] | $S\left( i \right)temp$*  $\boldsymbol{S}\left( \boldsymbol{i} \right)$ **< 0.5 = exploiters (E)**  $\boldsymbol{S}\left( \boldsymbol{i} \right)$ **≥ 0.5 = cooperators (C)**   - Step 1: calculate p(e) and p(c) - Step 2: Probability of interactions   $\boldsymbol{P} \left( \boldsymbol{C}\mathbf{-}\boldsymbol{C} \right)\mathbf{=}\boldsymbol{p}\left( \boldsymbol{c} \right)^{\boldsymbol{2}}$  $\boldsymbol{P} \left( \boldsymbol{C}\mathbf{-}\boldsymbol{E} \right)\mathbf{=}\boldsymbol{2}\mathbf{.}\boldsymbol{p} \left( \boldsymbol{c} \right)\mathbf{.}\boldsymbol{p}\left( \boldsymbol{e} \right)$  $\boldsymbol{P} \left( \boldsymbol{E}\mathbf{-}\boldsymbol{E} \right)\mathbf{=}\boldsymbol{p}\left( \boldsymbol{e} \right)^{\boldsymbol{2}}$   - Step 3 : Pay-off calculations (see pay-off matrix, supplementary table 4)   Original $S(i)$ of the selected cells, i.e., for cells which has current $S\left( i \right)^{'}\geq0.5,$were passed on to the next cycle   - Step 4: Sporulation probability calculation     $\boldsymbol{P}\left( \boldsymbol{i} \right)\mathbf{=}\boldsymbol{S}\left( \boldsymbol{i} \right)$ → 𝑆(𝑖)𝑡𝑒𝑚𝑝  → $S(i)temp$distribution | |
|  | Germination efficiency | $G\left( i \right)temp$*  $\mathbf{<}\boldsymbol{G}\mathbf{>}$ **=** $\boldsymbol{\varepsilon}\mathbf{.}$ $\frac{\boldsymbol{1}}{\boldsymbol{n}}\sum_{\boldsymbol{i}\mathbf{=}\boldsymbol{1}}^{\boldsymbol{q}} \boldsymbol{G}\mathbf{(}\boldsymbol{i}\mathbf{)}$**, ε = 0.5**  $\boldsymbol{P}\mathbf{(}\boldsymbol{i}\mathbf{) = <}\boldsymbol{G}\mathbf{>}$  $\to G\left( i \right)temp$ | |


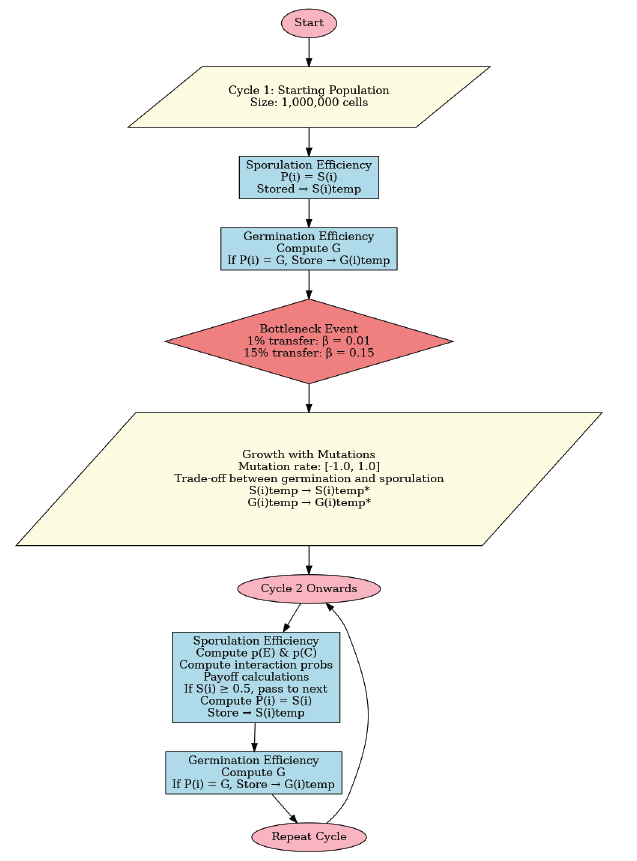

Supplement: S3 Table — The table and the flowchart describe the successive events included in the simulations of a simplified version of life cycle lab evolution, which include the alternating 10 cycles of sporulation, germination and growth phases. Mutations were allowed during the growth phase. Simulations were repeated for different combinations of γ [0,1] and epsilon [0,1] values. (DOCX) [file pbio.3003499.s010.docx]
