# Supplementary material for: Bottleneck size drives the evolution of cooperative traits in an aggregative multicellular myxobacterium
Source: PLoS Biol. 2026 Jan 6;24(1):e3003499. doi: 10.1371/journal.pbio.3003499 (PMC12773805; doi:10.1371/journal.pbio.3003499)
Supplement: S4 Table — During the sporulation phase, the cooperators (sporulation efficiency ≥0.5) and exploiters (sporulation efficiency <0.5) were allowed to interact and share public goods. The pay matrix is calculated as above for each type of allowed interaction. (DOCX) [file pbio.3003499.s011.docx]

S4 Table

| **Interactions**  **during sporulation** | Cooperator  $S\left( i \right)$ ≥ 0.5 | Exploiter  $S\left( i \right)$ < 0.5 |
| --- | --- | --- |
| Cooperator  $S\left( i \right)$ ≥ 0.5 | $\boldsymbol{K}\mathbf{=}\boldsymbol{\gamma}\mathbf{.}$ $\frac{\mathbf{1}}{\mathbf{2}} \sum_{\boldsymbol{i}\mathbf{=1}}^{\boldsymbol{m}} \boldsymbol{S}\mathbf{(}\boldsymbol{i}\mathbf{)}$  $\boldsymbol{\gamma}$ = [0,1] | $\boldsymbol{D}\mathbf{= <}\boldsymbol{Sc}\left( \boldsymbol{i} \right)\mathbf{>- <}\boldsymbol{Se}\left( \boldsymbol{i} \right)\mathbf{>}$  $<Sc\left( i \right)^{'}> = <Sc\left( i \right)>-\frac{D}{n}$**,**  $<S{e\left( i \right)}^{'}> = <Sc\left( i \right)>+\frac{D}{n}$*,*  $n=50$ |
| Exploiter  $S\left( i \right)$ < 0.5 | $\boldsymbol{D}\mathbf{= <}\boldsymbol{Sc}\left( \boldsymbol{i} \right)\mathbf{>- <}\boldsymbol{Se}\left( \boldsymbol{i} \right)\mathbf{>}$  $<Sc\left( i \right)^{'}> = <Sc\left( i \right)>-\frac{D}{n}$**,**  $<S{e\left( i \right)}^{'}> = <Sc\left( i \right)>+\frac{D}{n}$*,*  $n=50$ | $\boldsymbol{N}\mathbf{=}\boldsymbol{0}$ |
