# Supplementary material for: Bottleneck size drives the evolution of cooperative traits in an aggregative multicellular myxobacterium
Source: PLoS Biol. 2026 Jan 6;24(1):e3003499. doi: 10.1371/journal.pbio.3003499 (PMC12773805; doi:10.1371/journal.pbio.3003499)
Supplement: S1 Text — Description of social behaviors in M. xanthus. (PDF) [file pbio.3003499.s012.pdf]

Supplementary Information

Bottleneck size drives the evolution of cooperative traits in an aggregative multicellular myxobacterium

Jyotsna Kalathera[1], Prakhar Jaiswal[2], Neha Mandal[1], Vishwa Patel[1], Vishwesha Guttal[2], Sandeep Krishna[3], Samay Pande[1]

[1]Bacterial Ecology and Evolution group, Department of Microbiology and Cell Biology, Indian Institute of Science, Bengaluru, India

[2]Theoretical Ecology and Evolution laboratory, Centre for Ecological Sciences, Indian Institute of Science, Bengaluru, India

[3]Simons Centre for the Study of Living Machines, National Centre for Biological Sciences-TIFR, Bengaluru, India

Social traits of *M. xanthus*

Sporulation: Sporulation of *M. xanthus* is triggered by starvation and results in the formation of spore-filled fruiting bodies[1]. This process requires multiple contact independent signalling mechanisms such as quorum sensing [2,3], and death of a major fraction of cells die presumably to provide nutrients to the small fraction of cells that successfully develop into spores[4]. This process, thus, is susceptible to the evolution of non-cooperating individuals, as was demonstrated previously [5].

Predation: Predation by *M. xanthus* is mediated by both contact-dependent and independent antimicrobial mechanisms[6]. Contact-dependent killing mechanisms rely on the expression and use of secretion systems [7]. Contact-independent mechanisms are diverse and include the production of antibiotics, toxins, antimicrobial peptides, digestive enzymes and secondary metabolites[8]. Moreover, prey cells are digested extracellularly and hence, in addition to the antimicrobial substances that kill the prey, nutrients from the dead prey cells serve as public goods. It is easy to see how such a predatory strategy will rely on the high densities of the predator and should, therefore, show density dependence. Recent reports demonstrate that, indeed, predation by *M. xanthus* is density-dependent[9]. Each of the antimicrobial strategies listed above is costly to synthesize/express, thus, the cells that do not produce antimicrobial substances listed above stand to gain disproportionate benefit. Taken together, *M. xanthus* predation is driven by cooperative/synergistic interactions between individuals that are susceptible to exploitation.

Germination: Recent evidence suggests that *M. xanthus* germination efficiency increases as a function of the density of *M. xanthus* population[10]. Also, this process is susceptible to cheating behaviours by non-cooperating cells. Cheating, in this case, might be driven by the variants that disproportionately take advantage of diffused public goods.

Growth: Similar to previous report[11]**,** we show that the growth of *M. xanthus* is density dependent. Casitone is a complex media, and digesting extracellularly available autoclaved casitone media involves the secretion of digestive enzymes. Thus, the extracellular antimicrobial molecules as well as the digested prey are public goods that are available to the producers as well as the non-producers.

References

1. Reichenbach, H. & Dworkin, M. The Order Myxobacterales. in *The Prokaryotes: A Handbook on Habitats, Isolation, and Identification of Bacteria* (eds. Starr, M. P., Stolp, H., Trüper, H. G., Balows, A. & Schlegel, H. G.) 328–355 (Springer, 1981). doi:10.1007/978-3-662-13187-9_20.
2. Kaplan, H. B. & Plamann, L. A *Myxococcus xanthus* cell density-sensing system required for multicellular development. *FEMS Microbiol. Lett.* **139**, 89–95 (1996).
3. Shimkets, L. J. Intercellular signaling during fruiting-body development of *Myxococcus xanthus*. *Annu. Rev. Microbiol.* **53**, 525–549 (1999).
4. Wireman, J. W. & Dworkin, M. Developmentally induced autolysis during fruiting body formation by Myxococcus xanthus. *J. Bacteriol.* **129**, 798–802 (1977).
5. Velicer, G. J., Kroos, L. & Lenski, R. E. Developmental cheating in the social bacterium *Myxococcus xanthus*. *Nature* **404**, 598–601 (2000).
6. Berleman, J. E. & Kirby, J. R. Deciphering the hunting strategy of a bacterial wolfpack. *FEMS Microbiol. Rev.* **33**, 942–957 (2009).
7. Seef, S. *et al.* A Tad-like apparatus is required for contact-dependent prey killing in predatory social bacteria. *eLife* **10**, e72409 (2021).
8. Goldman, B. S. *et al.* Evolution of sensory complexity recorded in a myxobacterial genome. *Proc. Natl. Acad. Sci. U. S. A.* **103**, 15200–15205 (2006).
9. Muñoz-Dorado, J., Marcos-Torres, F. J., García-Bravo, E., Moraleda-Muñoz, A. & Pérez, J. Myxobacteria: Moving, Killing, Feeding, and Surviving Together. *Front. Microbiol.* **7**, 781 (2016).
10. Pande, S., Pérez Escriva, P., Yu, Y.-T. N., Sauer, U. & Velicer, G. J. Cooperation and Cheating among Germinating Spores. *Curr. Biol. CB* **30**, 4745-4752.e4 (2020).
11. Rosenberg, E., Keller, K. H. & Dworkin, M. Cell density-dependent growth of *Myxococcus xanthus* on casein. *J. Bacteriol.* **129**, 770–777 (1977).
